# Natural solutions for diabetes: the therapeutic potential of plants and mushrooms

**DOI:** 10.3389/fnut.2025.1511049

**Published:** 2025-07-04

**Authors:** Aleksandra Sknepnek, Dunja Miletić, Alena Stupar, Ana Salević-Jelić, Viktor Nedović, Aleksandra Cvetanović Kljakić

**Affiliations:** ^1^Faculty of Agriculture, Institute of Food Technology and Biochemistry, University of Belgrade, Belgrade, Serbia; ^2^Institute of Food Technology, University of Novi Sad, Novi Sad, Serbia; ^3^Faculty of Technology Novi Sad, University of Novi Sad, Novi Sad, Serbia

**Keywords:** antidiabetic properties, medicinal plants, medicinal mushrooms, bioactive compounds, extracts

## Abstract

Medicinal plants and mushrooms have been used for the prevention and treatment of various diseases since ancient times. For thousands of years, they have attracted significant interest due to their broad spectrum of biological activities and drug-like properties. Their continued use in traditional medicine has evolved alongside, and increasingly been supported by modern scientific research. Diabetes mellitus poses a serious global health, social and economic challenge and is among the most rapidly growing health issues of the 21st century. Type 2 diabetes mellitus (T2DM), which accounts for 90–95% of diabetes cases, is largely attributed to sedentary lifestyles, unhealthy diets and obesity. Herbal medicine has already played a key role in the development of antidiabetic drugs, as exemplified by the plant-derived origins of metformin. The development of new therapeutics or therapeutic adjuvants from natural sources offers several advantages over synthetic drugs, including improved safety profiles for long-term use, efficacy, affordability and reliance on renewable raw materials. This review highlights the potential of bioactive compounds from medicinal plants and mushrooms, discussing their mechanism of action, extraction techniques and their significance for the prevention, management and treatment of T2DM.

## 1 Introduction

Natural products, such as plants and mushrooms, have been used in the traditional medicine across various cultures since ancient times (1, 2). These natural derived therapies have long served in the prevention and treatment of many diseases, with evidence of their use even found in fossil records (1, 3). Today, their value extends beyond anthropological and archaeological significance. The World Health Organization (WHO) acknowledges traditional and complementary medicine as a valuable health resource with many potential applications in preventing and managing lifestyle-related chronic diseases (4). In addition to their role in traditional therapies, plants and mushrooms are widely consumed as health-promoting foods and are commonly used in the production of dietary supplements. These natural sources contain a diverse array of secondary metabolites with unique chemical structures, biological activities and drug-like properties (2, 5). This diversity has attracted increasing scientific interest. Modern research has further validated traditional medicine by supporting the selection and identification of medicinal plants, characterization of active compounds, and understanding of therapeutic principles (3, 6). Advances have also been made in standardizing preparation methods and dosage. The integration of modern scientific techniques with traditional knowledge-such as ethnomedicine and ethnobotany— has positioned natural products as important contributors to drug discovery (6). They continue to play a key role in the development of new nutraceuticals and pharmaceuticals. It is estimated that more than 40% of pharmaceutical drugs are derived from natural products originally used in traditional medicine (7).

The use of natural products as a foundation for developing new drugs and nutraceuticals offers several advantages, including the long-standing safety profile of bioactive compounds compared to synthetic alternatives. Furthermore, natural products are renewable sources of raw materials, enabling the extraction of unique and complex structures that may not be easily synthesized (1). Today, green extraction methods are increasingly used to unlock the therapeutic potential of these natural products. These environmentally friendly techniques focus on sustainability and safety, preserving the active ingredients while improving both yield and purity. By reducing harmful residues, green extraction makes the resulting extracts more suitable for health-related applications. The broad range of secondary metabolites obtained through these methods supports diverse biological activities and drug-like properties, reinforcing their value in modern research and development.

Diabetes mellitus (DM), commonly referred to as diabetes, is a chronic metabolic disorder affecting people worldwide. It is characterized by elevated blood glucose levels resulting from an imbalance in insulin—a hormone responsible for regulating blood sugar. This imbalance may be due to insufficient insulin production or the inability of cells to respond to it. If left undiagnosed or untreated, diabetes can lead to serious and potentially life-threatening health complications (8–10). According to the American Diabetes Association, diabetes is classified into four main types. Type 1 diabetes mellitus (T1DM) is an immune-mediated diabetes that occurs as a result of autoimmune destruction of pancreatic *β*-cells and usually leads to absolute insulin deficiency. Type 2 diabetes mellitus (T2DM), the most common form, is associated with obesity and involves either a gradual decline in insulin secretion by *β*-cells or a reduced sensitivity of the body’s cells to insulin (insulin resistance) (11).

T2DM accounts for approximately 90–95% of all diabetes cases. It is often referred to as non-insulin-dependent or adult-onset diabetes, although its exact etiology is unknown. Gestational diabetes is another form, diagnosed during the second or third trimester of pregnancy in individuals who did not previously have diabetes. The fourth category includes specific types of DM caused by a variety of underlying factors. These include genetic defects affecting *β*-cell function or insulin action, as well as diseases of the exocrine pancreas such as cystic fibrosis and pancreatitis that can contribute to the development of diabetes. Additionally, this group covers drug- or chemical-induced DM or DM caused by viral or other infections, as well as rare forms of immune-mediated diabetes and other genetic syndromes sometimes associated with diabetes (11).

According to the International Diabetes Federation (IDF), diabetes is one of the fastest-growing global health challenges of the 21st century. Among adults aged 20 to 79, the estimated number of people living with diabetes rose from 151 million in 2000 to 425 million in 2017, 537 million in 2021, reaching 589 million in 2024 (11.1% of the adult population). Without effective interventions this number is projected to increase to 643 million by 2030 and 783 million by 2045 posing a serious public health concern across all regions. Diabetes not only affects individual health but also places a substantial social and economic burden on healthcare systems. In 2024 alone, diabetes was responsible for 3.4 million deaths (9.3% of all causes global deaths) and an estimated USD 1.015 trillion in healthcare spending. This accounts for 12% of worldwide healthcare costs and 338% increase since 2007 (12).

The near pandemic prevalence of T2DM which accounts for over 90% of all diabetes cases is largely attributed to modern lifestyles characterized by physical inactivity, poor dietary habits and rising obesity rates. This growing health crisis underscores the urgent need for effective treatment, prevention strategies, and education to support better health outcomes. Biochemical studies have revealed correlation between obesity and T2DM with insulin resistance. However, not all individuals with obesity and insulin resistance develop hyperglycemia. In many cases, pancreatic *β*-cells can produce and release a sufficient concentration of insulin that overcomes their reduced efficiency and maintain normal blood glucose levels. On the other hand, high blood glucose levels are not a mitigating factor in patients with pancreatic *β*-cell dysfunction, and they are at high risk of developing T2DM (13).

Insufficient insulin secretion not only disrupts blood glucose regulation but also contribute to the development of serious complications, including cardiovascular disease, blindness, kidney failure and limb amputations (14). Importantly, T2DM is considered largely preventable and manageable through health dietary habits and lifestyle changes (8, 10). In an effort to prevent and control T2DM, there has been renewed interest in natural products traditionally known for their therapeutic properties. Medicinal plants and mushrooms, in particular, are being explored as sources of bioactive compounds with antidiabetic potential. These natural sources may serve as ingredients in functional foods or nutraceuticals aimed to prevent diabetes and its complications. Advancements in the identification of plant and mushroom species, along with analyses of their bioactive compounds, phytochemistry, pharmacology, and toxicology provide a strong foundation for the development of new, safe and cost-effective antidiabetic agents (8, 15). The growing interest in this field is evident from a search of the PubMed database (16). The keywords *plant* and *diabetes* yielded 22,201 publications, including 1,103 clinical studies, while *mushrooms* and *diabetes*, resulted in 530 publications, with 7 clinical trials.

The objective of this review is to provide a comprehensive analysis of the role of phytotherapy and mycotherapy in the prevention, management, and treatment of T2DM. This review explores a range of bioactive compounds derived from medicinal plants and mushrooms, discussing their mechanisms of action, extraction methods and therapeutic potential. By integrating insights from traditional knowledge with findings from scientific research, this review aims to illustrate how these natural solutions can complement existing diabetes management approaches and contribute to improved health outcomes.

## 2 The role of naturally derived bioactive compounds in type 2 diabetes mellitus treatment

The development of natural antidiabetic nutraceuticals and therapeutics from plants offers a promising strategy for improving physiological conditions and managing diabetes. Plants from different regions around the world, along with their extracts and isolated compounds have been extensively studied for their antidiabetic properties. These plant-derived substances exhibit a variety of mechanisms of action in diabetes control, including the inhibition of enzymes that metabolize carbohydrates to reduce glucose absorption in the intestine, suppression of sodium-glucose co-transporter; enhancement of hepatic enzymatic activity, and improvement of pancreatic *β*-cell function. Additionally, many plant extracts demonstrate antihyperglycemic, hypolipidemic and antioxidant effects. Beyond glucose regulation, plant-based therapies may also address diabetes-related complications. Reported benefits include neuroprotective and immunomodulatory effects, protection against diabetic nephropathy, a prevention of cardiovascular complications and promotion of diabetic wound healing (15).

Numerous plant-derived extracts and isolated compounds have shown proven antidiabetic potential. Notable examples includes berberine derived from spices, ginsenosides, curcumin and capsaicin, sterols derived from plants, phenolic compounds derived from herbs, tea, fruits and vegetables (catechins, phenolic acids, anthocyanins, resveratrol, isoflavonoids, flavanones); terpenoids and alkaloids derived from herbs (10, 15, 17). Overall, these studies underline the great potential of natural plant-based products as effective and affordable sources for the development of new therapeutics or therapeutic adjuncts.

Medicinal mushrooms, much like medicinal plants, have transitioned from traditional therapy and natural health food to valuable components of modern medicine and functional food. They are increasingly recognized as a source of various biologically active compounds that have offer a range of nutritional and therapeutic benefits, including notable hypoglycemic and antidiabetic effect, making them a promising source of nutraceuticals and pharmaceuticals. From a nutritional standpoint, edible mushrooms are well-suited for diabetic diets due to their high fiber, protein and mineral content coupled with low fat and energy density. Research on the antidiabetic properties of mushrooms by examining both their fruiting bodies and cultured mycelia, identified several mechanisms for controlling and reversing diabetes. These include lowering serum glucose levels, inhibiting carbohydrate-metabolizing enzymes, enhancing pancreatic *β*-cells function, increasing insulin secretion, prebiotic activity, exerting antioxidant effects, suppressing oxidative stress, and supporting diabetic wound healing. A wide variety of bioactive compounds found in mushrooms have been linked to these effects. These include oligosaccharides, glucan-rich polysaccharides, dietary fibers, polysaccharide-peptide complexes, terpenoids, sterols and phenolic compounds – each contributing to the mushrooms’ antidiabetic potential (8, 18).

Commercial mushroom extracts are available on the market, containing ingredients that support healthy blood glucose levels. One notable example is SX-fraction^®^ developed by Mushroom Wisdom. This product is derived from the fruiting body of the maitake mushroom (*Grifola frondosa*) and contains a bioactive glycoprotein. The composition and extraction process of SX-fraction^®^ are protected under US patent no. 7,214,778 (19).

Sodium-glucose co-transporter (SGLT) inhibitors based on phlorizin, a plant-based active ingredient, have made a significant contribution to modern antidiabetic therapy. Phlorizin, a dihydrohalcon first isolated from the bark of apple trees in 1835, inhibits both SGLT1 and SGLT2. Notably, SGLT2 inhibition is one of the key mechanisms for glucose homeostasis. Due to its inhibitory effects against SGLTs and certain critical limitations, phlorizin has been the subject of numerous studies aimed to find an analog with improved SGLT2 selectivity, bioavailability and stability. Thus, dapagliflozin was developed by AstraZeneca and Bristol Myers Squibb Company as a selective SGLT2 inhibitor for the treatment of T2DM, effectively lowering plasma glucose levels and glycosylated hemoglobin and improving glycemic control and body weight reduction (17, 20). Dapagliflozin has been approved and marketed for the treatment of T2DM by both the European Union (21) and the US Food and Drug Administration (FDA) (22). Since then, numerous other SGLT inhibitors have been derived from phlorizin and approved by regulatory agencies such as the FDA, the European Medicines Agency (EMA) and health authorities in Japan. These agents are administered orally and are either in clinical use or undergoing clinical trials for diabetes treatment (20). The valuable role of ethnomedicine in antidiabetic drug development is also exemplified by the history of metformin (1,1-dimethylbiguanide hydrochloride; Met), a widely used oral hypoglycemic agent. Its origin traces back to the traditional medicinal use of *Galega officinalis*, which contains guanidine and its derivatives – compounds that served as the foundation for metformin’s synthesis (1, 23). Currently, oral antidiabetic medications are categorized into five main groups: insulin secretagogues, insulin sensitizers, biguanides, *α*-glucosidase inhibitors and dipeptidyl peptidase-4 inhibitors (DPP-4 inhibitors). Among these, α-glucosidase inhibitors are most commonly used to lower blood sugar after digestion. Enzyme inhibitors such as acarbose, voglibose and miglitol are used in clinical practice for the treatment of T2DM and as adjunct therapies in the treatment of T1DM (24). *α*-glycosidase inhibitors can be synthetically produced or extracted from animals, plants and microorganisms, or identified as microbial metabolic products (24).

## 3 Sources of antidiabetic compounds: plants and mushrooms

Bioactive compounds derived from natural sources, particularly plants and mushrooms, play a pivotal role in the development of antidiabetic therapeutics. Both plants and mushrooms contain a diverse array of secondary metabolites that exhibit significant pharmacological activities, including the regulation of blood glucose levels and the mitigation of diabetes-related complications. The complexity of their chemical compositions and the various mechanisms through which they exert their effects underscore their potential as effective agents in diabetes management. Accordingly, this section explores the major bioactive compounds from both plants and mushrooms, highlighting their therapeutic properties, modes of action, and the implications for their use in treating and preventing diabetes.

### 3.1 Plants

**Figure 1:**
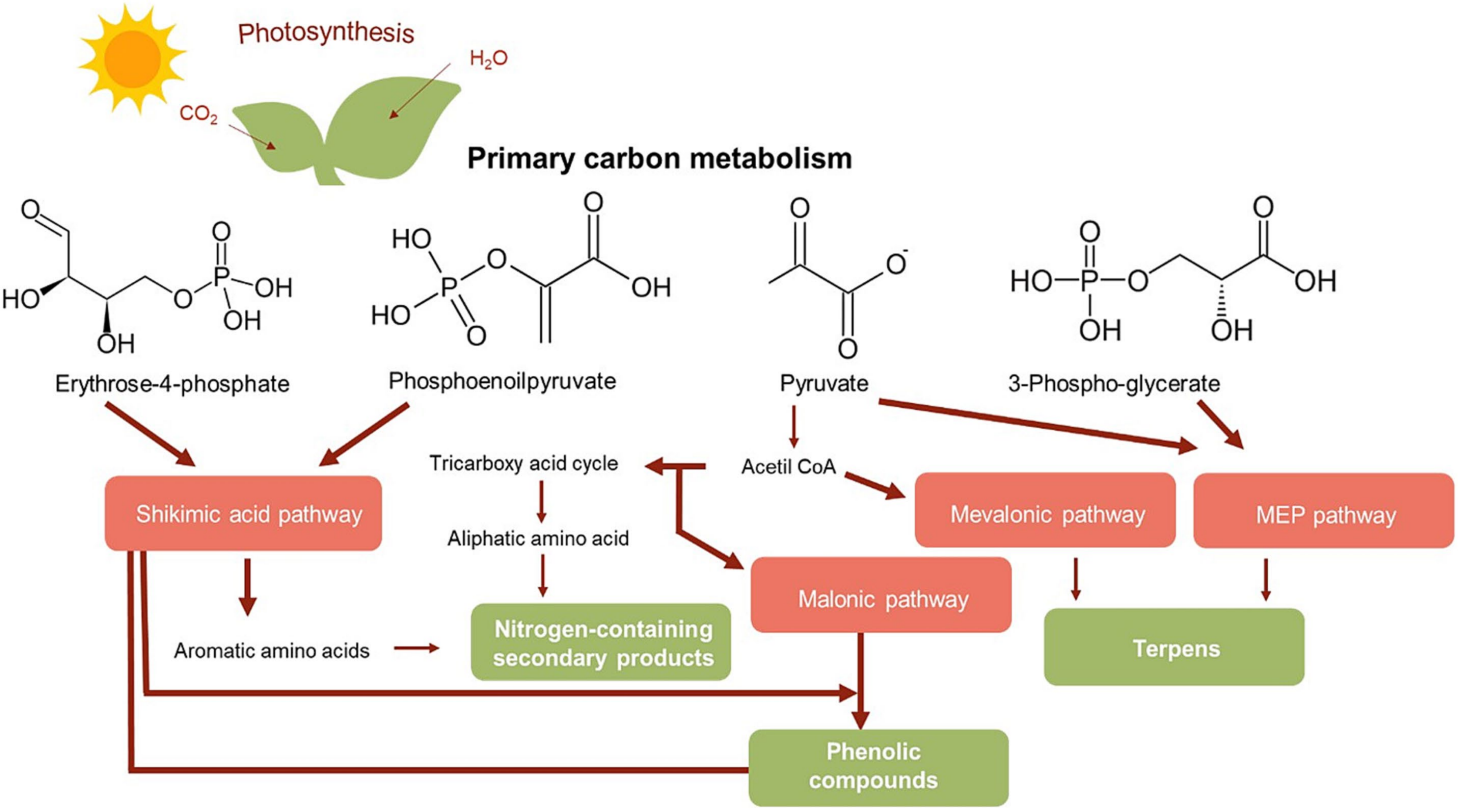
Simplified representation of the main metabolic pathways and biosynthesis of plant secondary metabolites. Reproduced from (25) with permission from Springer Nature.

Bioactive compounds of plant and mushroom origin hold a significant place in modern pharmacology. Plants, characterized by their complex chemical composition, exhibit a broad spectrum of pharmacological activities. The therapeutic effects of these organisms are primarily based on the chemical structure of their constituents, their pharmacodynamics, and bioavailability. Among the most important contributors to these effects are secondary metabolites. Secondary metabolism in plants is a direct continuation of primary metabolism, in which more than 200,000 structurally different compounds can be formed. These compounds play essential roles in plant survival and interactions with the environment. They are synthesized in different parts of the plant via pathways that typically involve two phases. The first phase corresponds to primary metabolism, while the second phase is less clear and is based on intermediates formed during the first. The final metabolites vary depending on the specific enzymes and regulatory mechanisms present in different plant species (25). Due to the complexity of these biogenetic metabolic pathways and the presence of many unidentified enzymes, chemical synthesis of secondary metabolites is challenging (Figure 1). As a result, current pharmacological use of these compounds focuses on their isolation from natural sources.

The large number and structural diversity of secondary metabolites make their classification difficult. Today, several subdivisions of this large group of compounds exist. However, there are no clear boundaries between these classes, and overlapping is frequent. The two most important classifications are certainly those based on their chemical structure or their biogenetic origin. Regardless of classification, all secondary metabolites share a high degree of biological activity, which is preserved even after extraction from the plant matrix. Key biological activities include antioxidant, antimicrobial, cytotoxic, antifungal, and anti-inflammatory effects, with particular emphasis on their potential therapeutic roles in diabetes management (26, 27).

According to ethnopharmacological data, over 800 plant species are used in the treatment of diabetes. Notably, several plant-derived compounds have demonstrated significant antidiabetic properties. For instance, flavonoids such as quercetin and kaempferol – found in onions and apples – enhance insulin sensitivity; alkaloids like berberine, extracted from *Berberis vulgaris*, effectively lower blood glucose levels; phenolic acids, such as chlorogenic acid present in coffee, improve glucose metabolism; terpenoids like ginsenosides from ginseng support insulin sensitivity; and polysaccharides found in soluble fibers from oats aid in glycemic regulation (28–31). Many of them are used in the preparation of extracts, tinctures and other herbal formulations, which may be applied at different stages of the disease. Others are valued for their preventive potential, particularly among individuals with a genetic predisposition to diabetes. Their ability to regulate blood sugar levels positions them as promising agents for controlling the progression of the disease and its possible complications. Harnessing their potential offers modern science valuable avenues for addressing limitations of conventional medicines and therapies, including gastrointestinal disorders, possible toxicity, etc.

### 3.2 Mushrooms

Mushrooms have been consumed for centuries as both food and a valuable source of micro- and macronutrients due to their high nutritional value. Although approximately 14,000 species of mushrooms are known to date, only about 25 of them are commonly consumed in the human diet and even fewer are commercially cultivated. About 700 species exert various pharmacological activities and can be used in the form of fruiting bodies, mycelia or different extracts for the prevention and treatment of various diseases (32, 33). In the context of diabetes control and prevention of diabetes, mushrooms are a good dietary choice due to their low-calorie content and low glycemic index. In addition, mushrooms contain bioactive compounds that may have antidiabetic properties and therefore be used for their isolation and application.

In addition to triterpenoids, alkaloids and phenolic compounds, among the most important bioactive compounds in mushrooms with antidiabetic effects include polysaccharides, glycoproteins, and proteins (34–36). The biological activity of mushroom extracts or individual compounds depends on several factors, including the mushroom strain, cultivation method, cultivation conditions, stage of maturity and the extraction and purification methods used (37). Polysaccharides from medicinal mushrooms can be isolated from the fruiting bodies, mycelium or culture broth when cultivated in submerged conditions. They can be obtained by extraction, separation and purification in form of polysaccharides, polysaccharopeptides and polysaccharide-proteins (38, 39). During mushroom extraction, the cell wall is ruptured and the polysaccharides and glycoproteins are extracted from the outer layer of the cell wall, while the glucans can be obtained from the inner, middle and outer layers. Polysaccharides are high molecular weight polymers that are indigestible and have significant bioactivity, including antioxidant, antitumor and immunomodulatory activity. The structural features of these macromolecules are of great importance for their bioactivity. Key features include the presence of non-sugar compounds (e.g., proteins, sulfate groups), the chain conformation, the presence of double or triple helices and the degree of branching (40).

## 4 Bioactive compounds and their mechanisms of antidiabetic action

### 4.1 Polyphenols from plants and mushrooms in antidiabetic therapy: biosynthesis, mechanisms and therapeutic potential

Polyphenols are a crucial group of secondary metabolites, characterized by an aromatic nucleus with at least one hydroxyl group. They include a wide range of compounds that can be classified as: simple phenols (C6), hydroxybenzoates (C6-C1), acetophenones and phenylacetates (C6-C2), coumarins, chromones, hydroxycinnamates, phenylpropenes (C6- C3), naphthoquinones (C6-C4), xanthones (S6-S1-S6), stilbenes and anthraquinones (S6-S2-S6), flavonoids (S6-S3-S6), lignans (S6-S3)2, biflavonoids (S6-S3-S6)2, catechol melanini (S6)n, lignins (S6-S3)n and condensed tannins (S6-S3-S6)n which are the most numerous, exceeding 6,000 compounds (41). The biosynthesis of polyphenols occurs through specific metabolic pathways. One of the main routes is the shikimate-arogenate pathway, which leads to the production of phenylpropane compounds and aromatic amino acids such as phenylalanine, tyrosine, and tryptophan. This pathway is crucial not only for amino acid biosynthesis but also for generating important substances like lignin (42). Another significant pathway, known as the acetate-malonate pathway, leads to the production of various phenolic compounds, including quinones, anthraquinones, chromones, isocoumarins, flavonoids, stilbenoids, styrilpyrones, etc. Some polyphenols arise from the integration of both pathways, illustrating the complexity of their biosynthetic processes. The biosynthesis of polyphenolic compounds is endogenously controlled during plant development (43, 44) and is also influenced by exogenous factors such as light, temperature, damage and other stress factors (45). In addition to plants, phenolic compounds are also synthesized in fungi, including mushrooms and yeasts, through similar biosynthetic routes – most notably the shikimate pathway. The predominant phenolic compounds in mushrooms include hydroxybenzoic acids (gallic acid, p-hydroxybenzoic acid, protocatechuic acid, etc.), hydroxycinnamic acids (ferulic acid, p-coumaric acid, etc.) as well as various other phenolic derivatives. The biosynthesis of these compounds in mushrooms is also influenced by endogenous and exogenous factors such as temperature, light, interaction with other microbes and oxidative stress (46).

Plants and mushrooms represent rich natural reservoirs of polyphenolic compounds, where these bioactives serve important roles in ecological defense and physiological regulation. Polyphenolic compounds are gaining increasing attention for their positive health effects and are commonly incorporated into functional foods. In the context of diabetes, polyphenols exhibit notable antidiabetic activity through various mechanisms, including antioxidant activity, antilipoxidation, enzyme inhibition (*α*-amylase and glucosidase), aldose reductase modulation, antiglycation, and gene regulation (47). Their antidiabetic effects primarily stem from their ability to neutralize free radicals, which is essential for managing blood sugar levels. Oxidative stress has a significant impact on the development and progression of DM, and its influence is associated with organ damage, particularly kidney and liver damage (36). Oxidative stress is defined as the excessive production of reactive oxygen species (ROS) that exceeds the capacity of the organism’s antioxidant defense mechanisms including both, enzymatic and non-enzymatic antioxidants to neutralize them. Chronic hyperglycemia in DM patients, as well as in cancer and neurodegenerative diseases, contributes to persistent oxidative stress (34). As a result, the body’s endogenous antioxidant defense system is unable to protect the organism from the undesirable effects of ROS by producing sufficient amounts of scavengers such as superoxide dismutase (SOD), catalase (CAT) and glutathione peroxidase (GSHPx). When ROS react with polyunsaturated fatty acids, they can cause lipid peroxidation and consequently damage membrane function and activity (48). In the treatment of DM, a combination of drugs and therapies is usually required to maintain optimal blood glucose levels. One of the complementary DM treatments targets both blood glucose levels and oxidative stress. In this context, plants, mushrooms and their extracts, as proven antioxidants, can be a good alternative source of bioactive components to improve blood glucose control in conjunction with conventional drugs (49–53). Studies indicate that both oral and intravenous administration of polyphenols can effectively lower blood glucose levels by reducing glucose absorption in the intestine, enhancing uptake in peripheral tissues, and limiting reabsorption in the kidneys. Various polyphenols such as anthocyanins, caffeic acid, catechin, quercetin, isoferulic acid and epigallocatechin have a positive effect on the treatment of diabetes (54). For example, a study by Junejo et al. (55) showed that apigenin derivatives have a significant antidiabetic activity and can help protect against diabetic complications. At the same time, a study by Pandey and Rizvi (56) showed that quercetin can reduce lipid peroxidation and inhibit cellular oxidation in diabetes. In addition, polyphenolic and flavonoid components protect and regenerate pancreatic *β*-cells and inhibit lipid peroxidation and other pro-oxidative processes.

Compared to flavonoid aglycones, glucoside molecules are more active in inhibiting *α*-amylase and α-glucosidase. For example, apigetrin (apigenin-7-O-glucoside) can inhibit 50% of *α*-amylase at a concentration of less than 0.2 mM, while luteolin-7-O-glucoside achieves 100% inhibition at a concentration of 5 mg/mL (57).

*In vitro* assays showed that mushroom phenolic compounds can also inhibit the activity of both α-amylase and α-glucosidase enzymes. Among the identified gallic acid, protocatechuic acid, epigallocatechin gallate, caffeic acid, naringin, resveratrol, kaempferol, and biochanin-A are highlighted as the compounds responsible for the enzyme inhibitory activity (58). The extracts of *Ganoderma pfeifferi* showed good protective effect on liver biochemical parameters of alloxan-induced diabetic mice, which might be correlated with the presence of gallic acid, due to the presence of hydroxyl groups, as well as flavonoids, which affect oxidoreductase enzymes by either inhibiting or activating them (34). The high total phenolic content, total flavonoid content and the lowest IC_50_ value for DPPH of the ethanol extract of *Ganoderma lucidum* correlated with the most significant *α*-glucosidase inhibitory activity among the eight species tested. The results proved that the reduction of oxidative stress through the action of phenolic compounds such as flavonoids may be beneficial for the health of diabetic patients (59).

Polyphenols are also capable of inhibiting aldose reductase – an enzyme involved in the polyol pathway that becomes hyperactive under hyperglycemic conditions. This leads to excessive sorbitol accumulation, causing osmotic stress and damage to nerves and kidneys tissues causing nephropathy, retinopathy and cataract development. When blood glucose levels are high, more than 30% of glucose is directed into this metabolic pathway, leading to an accumulation of sorbitol in the tissues. Under normal conditions, less than 3% of glucose is metabolized through this pathway (60–62). This accumulation of sorbitol can cause diabetic complications such as cataracts, retinopathy, neuropathy and nephropathy due to the creation of localized hyperosmotic conditions. Therefore, aldose reductase is considered an important target for the treatment of these diabetes-related complications. Flavonoids like luteolin, kaempferol, and epicatechin have shown effective inhibition of this enzyme. Additionally, some mushrooms with a high content of phenolic compounds can inhibit the activity of aldose reductase (61, 62). Bioactive compounds such as (+)-catechin/(−)-epicatechin, kaempferol, luteolin and β-glucogalin have shown the ability to inhibit this enzyme (63). The blood sugar-lowering effect of polyphenols is important in connection with the reduction of AGE values (advanced glycosylated end products) at high blood glucose levels. By supporting insulin action, polyphenols help lower blood glucose and improve metabolic health. Insulin not only supports the uptake of glucose into the cells, but also inhibits glucagon secretion and regulates glycogen production. Certain phytochemicals, such as berberine, can help lower blood sugar levels by mimicking insulin action. In this way, these compounds can help to reduce insulin resistance and alleviate the symptoms of diabetes. Curcumin also affects glucose metabolism by converting glucose to glucose-6-phosphate, which lowers glucose levels in cells and blocks the migration of the glucose transporter type 4 (GLUT4) proteins across the membrane (47).

Polyphenolic substances have also been shown to reduce oxidative stress by altering gene expression along these signaling pathways and increasing insulin production. Studies have shown that resveratrol (0.1 M) and curcumin (1 ppm) supplementation increases insulin secretion and improves *β*-cell function by inhibiting the gene expression of phosphodiesterase (Pde3b, Pde8a and Pde10a) through activation of the cAMP signaling pathway in human (HP62) and mouse pancreatic β-cells (64).

### 4.2 Terpenes and terpenoids from plants and mushrooms in antidiabetic therapy: biosynthesis, mechanisms and therapeutic potential

Terpenes represent a large and highly diverse group of secondary metabolites, primarily lipophilic, encompassing around 30,000 compounds. They are one of the main components of higher plants (genera of the families Apiaceae, Asteraceae, Lamiaceae, Myrtaceae, Pinaceae, Rosaceae, etc.), but can also be synthesized by animals and microorganisms. Despite their large number, all terpenoid components consist of isoprene units (5C) linked together according to the “head-tail” principle (41), and depending on the number of isoprene units, all terpenes are divided into several classes (Table 1).

**Table 1:** Classes of terpenes.

Number of C-atoms	Number of isoprene units	Name	Precursor
10	2	Monoterpenes	GPP
15	3	Sesquiterpenes	FPP
20	4	Diterpenes	GGPP
25	5	Sisterpenny	GFPP
30	6	Triterpenes	Squalene
40	8	Tetraterpenes	Phytoene
>40	N	Polyprenols	GGPP+(C_5_)n

Terpenes, although typically associated with plants, also represent a significant class of secondary metabolites produced by mushrooms. These compounds contribute to the distinctive aromas, defense mechanisms, and therapeutic properties of both plants and fungi. Terpenoids derived from these sources exhibit potent biological activities, including antimicrobial, anti-inflammatory, antioxidant, and anticancer effects, thanks to their ability to easily penetrate cell membranes and disrupt cellular processes (65).

Treating and controlling diabetes without side effects remains one of the greatest scientific challenges, and one of the potential solutions to this goal could be terpene-rich medicinal plants and mushrooms. Historically, they have been widely utilized by various cultures, and today, some of the mechanisms underlying the action of terpenes are understood. The mechanisms of antidiabetic action of this unique class of compounds may be diverse and include: insulin-mimetic action, ability to inhibit specific enzymes (*α*-amylase, glucosidase, aldose reductase), antioxidant activity, and the ability to regulate hypo/hyperglycemia (66). *In vivo* studies on mice have shown that the terpene compound stevioside and its derivatives can mimic insulin. This is because they can facilitate glucose breakdown, i.e., increase glycolysis (the production of new glucose), thereby successfully regulating blood glucose levels. This is closely related to their effect on relevant genes or their ability to inhibit certain enzymes in the liver (67). Moreover, this terpene and its derivatives can also act on the glucose transport system in skeletal muscle, as studies in rats have shown (68).

Terpenes exhibit a strong tendency to inhibit *α*-glucosidase and amylase. Individual components (hyptadienic acid, isolated from *Potentilla fulgens*, pentacyclic triterpene acetates isolated from the stem bark of *Fagara tessmannii*, corosolic acid isolated from the leaves of *Lagerstroemia* species) show a strong inhibitory potential towards α-glucosidase (69–71). Some of them even demonstrate a stronger inhibitory capacity than the control substance acarbose. Studies have shown that chamomile essential oils obtained by various technological processes have a greater affinity to inhibiting amylase than glucosidase (72). In contrast, terpene-rich chamomile extracts show a better tendency to inhibit glucosidase. Interestingly, lipophilic chamomile extracts show higher activity than chamomile essential oils, which is closely related to the synergistic effect of terpenes with non-terpenic compounds (72). However, *in vitro* studies have shown that the oils exhibit a significantly stronger effect compared to the extracts. Oxygenated sesquiterpenes, which are characterized by a strong antioxidant activity, have been identified as the main components in chamomile essential oils. Studies have shown that these oils express a high degree of neutralization of free radicals, with this effect being 5–7 times more pronounced for ABTS radicals than for DPPH radicals (72).

Research has shown that a large number of different mushrooms terpenoids inhibit *α*-glucosidase activity, and there is a clear relationship between their structure and activities (73–75). For example, the triterpenes of *Ganoderma resinaceum* exhibit *α*-glucosidase inhibitory effect due to the presence of a C-24/C-25 double bond, which is enhanced by the presence of a carboxylic group at C-26 and a hydroxyl group at C-15 (74). By comparing three *Ganoderma lingzhi* terpenoid compounds (ganoderol B, ganoderiol F and ganodermanontriol) researchers concluded that the inhibition of *α*-glucosidase activity depends on the presence of the hydroxyl at C-3 and the double bond (△^24(25)^) (76). The triterpenoids ganoderolactone B, D, E and ganoderoid A from *G. lucidum* have been shown to have an antidiabetic effect. The structure of the triterpenoids of *Ganoderma* spp., specifically the side chains, play an important role in α-glucosidase inhibitory activity (34). Mushroom triterpenes can also act as insulin sensitizers. For example, lanostane-type triterpenes like pachymic acid and dexydrotrametenolic acid, isolated from *Poria cocos* mushroom, show the ability to activate PPAR-*γ*, a ligand-activated transcription factor that causes insulin sensitization *in vitro* (77).

Additionally, various plant terpenes can inhibit aldose reductase, an enzyme linked to diabetic complications such as cataracts. This highlights the potential of various plant terpenes in managing diabetic complications by targeting aldose reductase activity, which is crucial for the prevention and treatment of diseases such as cataract associated with diabetes. In particular, various triterpenes and diterpenes from different plant sources have shown promise in this regard. For instance, extracts of several *Salacia* species such as *Salacia reticulate* (78–82), *Salacia oblonga* (83) and *Salacia chinensis* (synonyms S*alacia prinoides*) (84, 85) have shown hypoglycemic effects in rats orally loaded with sucrose and maltose. They also exibit inhibitory activities against *α*-glucosidases (e.g., sucrase, maltase and isomaltase) and aldose reductase in the rat lens, as well as hepatoprotective effect on CCl_4_-induced liver damage, antioxidant activity and anti-obesity effects. The monocyclic monoterpene D-limonene inhibits aldose reductase activity, which has been shown to delay the development of diabetic cataracts in streptozotocin (STZ)-induced diabetic rats (86). Terpenes are characterized by both hyper- and hypoglycemic effect, which have been demonstrated in several clinical studies (87, 88). A clinical study with the fruit extract of *Capparis spinosa* (capers) in patients with T2DM showed a significant reduction in blood glucose levels and glycosylated hemoglobin (89). In addition, olibanum gum resin from *Boswellia* trees significantly lowered fasting blood glucose, HbA1C, insulin, cholesterol, LDL and triglycerides in patients with T2DM (90). The methanolic extract of *Ginkgo biloba* inhibits pancreatic lipase with significant efficacy (IC_50_ = 16.5 μg/mL), contributing to its hypolipidemic and lipid-lowering effects. The terpene trilactones of *G. biloba* play a key role in this inhibition (91). Dehydroabietic acid, a diterpene, improves glucose and triglyceride levels in obese diabetic mice by modulating inflammatory markers and reducing the accumulation of macrophages in adipose tissue (92).

### 4.3 Alkaloids from plants and mushrooms in antidiabetic therapy: biosynthesis, mechanisms and therapeutic potential

Alkaloids are a structurally diverse class of nitrogen-containing secondary metabolites produced by various organisms, including plants, fungi (notably mushrooms), and certain bacteria, known for their potent pharmacological and toxicological effects, which underpin their medical applications. They are estimated to occur in 14–20% of plant species and in some mushrooms, where they contribute to the bioactive profile of fruiting bodies and mycelia (93).

These compounds are biosynthesized primarily through amino acid-derived pathways and serve essential ecological roles, such as defense against herbivores and pathogens. Although the biosynthesis of alkaloids and their role in plants is not fully understood, it is assumed that their ecological importance lies in the fact that poisonous plants deter animals from consumption (94). In both plants and mushrooms, alkaloids are typically present in conjugated forms – salts, esters, or amides – and exhibit a broad spectrum of pharmacological activities, including analgesic, antimicrobial, anticancer, neuroactive, and antidiabetic effects. Their therapeutic properties are largely influenced by their complex chemical scaffolds, such as isoquinolines, indoles, tropanes, quinolines, and pyridines (95, 96).

In the context of DM, alkaloids from both botanical and fungal origins have demonstrated promising antihyperglycemic properties. These effects are mediated through multiple mechanisms, including the inhibition of key digestive enzymes (*α*-glucosidase and α-amylase), suppression of hepatic glucose production, and modulation of glucose uptake and insulin sensitivity, while certain alkaloids inhibit aldose reductase. Interestingly, some plant-derived alkaloids have shown greater inhibitory activity than synthetic agents such as zopolrestat and tolrestat (27).

Alone or in combination with other natural agents, many alkaloids exhibit high degrees of activity and potential for the treatment of diabetes. Although alkaloids have some degree of toxicity, research shows that their benefits often outweigh the drawbacks. One challenge in developing new alkaloid-based drugs for diabetes treatment is their limited ability to bind to receptors involved in glucose homeostasis. Nevertheless, continued research into the mechanisms of action of alkaloids remains crucial for the development of new therapeutics for diabetes. Today, several alkaloid-based antidiabetic agents have been developed, such as miglitol, a derivative of deoxynojirimycin, which is used to lower blood glucose levels (97) and has been approved by the Food and Drug Administration (FDA).

Like most natural bioactive plant compounds, their effect is primarily attributed to their ability to inhibit the excessive activity of digestive enzymes involved in the catabolism of carbohydrates. Alkaloids considered promising for antidiabetic therapy typically exhibit the ability to inhibit *α*-glucosidase and moderate to weak inhibition of α-amylase activity (98). In addition to these two enzymes, the alkaloids have also demonstrated the ability to inhibit human aldose reductase, an enzyme whose overactivity is associated with diabetic neuropathy (99, 100). Compared to certain synthetic inhibitors such as zopolrestat and tolrestat, some alkaloids, especially isoquinoline and bis-isoquinoline, have shown superior aldose reductase inhibitory activity (101).

A study conducted by Liang et al. (102) revealed plants from the Moraceae family possess significant antidiabetic potential. Six alkaloids, including piperidine, pyrimidine and pyrazine derivatives, extracted from the leaves of *Morus atropurpurea* have been reported as an inhibitors against *α*-glucosidase (103). Similarly, polyhydroxylated and tropane alkaloids isolated from the leaves of *Morus bombycis* and *Morus alba* have also shown inhibitory effects (N-methyl-DNJ, 2-O-*α*D-galactopyranosyl-DNJ, 1,4-dideoxy-1,4-imino-D-arabinitol, 2-O-*β*-D-glucopyranosyl-D-arabinitol and lα,2β,3α,4β-tetrahydroxynor-tropane) (104, 105). One of the most potent compounds, plicatain, was isolated from *Chrozophora plicata*, and exhibited strong α-glucosidases inhibition with an IC_50_ value of 27.80 μm (106). Strong antidiabetic activity has also been reported for alkaloids from species belonging to the families Euphorbiaceae, Apocynaceae, Ranunculaceae, Bignoniaceae and Campanulaceae, Rutaceae, Abaceae, Portulacaceae, Acanthaceae and Piperaceae. Several of these compounds are currently under clinical investigation for their efficacy in the treatment of T2DM. These studies are focused on their ability to reduce blood glucose levels and HbA1c and combat dyslipidemia. For example, berberine and trigonelline are among the alkaloids being explored for these effects (107–109).

In addition to the pure compounds, alkaloid-rich plant extracts also exhibit significant potential to treat diabetes. This is particularly important given that the preparation of extracts is generally simpler and more cost-effective than the isolation and purification of individual pure compounds. Clinical studies have demonstrated promising results using alkaloid-rich extract from the root of *Sida cordifolia* in the treatment of diabetic polyneuropathy. An *in vivo* study by Zhang et al. (110) using extracts of *Litsea glutinosa* bark in diabetic laboratory mice also confirmed the extracts’ antidiabetic and antihyperlipidemic potential. Oral administration of the extracts for 4 weeks at varying concentrations reduced inflammation, increased oral glucose tolerance and serum lipase activity, indicating their beneficial effects on metabolic disorders. Research conducted by Sharma et al. (111) also showed positive effects of *Capparis decidua* extracts in diabetic mice. After 28 days of administration, blood glucose levels in diabetic mice decreased significantly, as did total cholesterol and triglycerides. The glycogen content in the liver and muscle tissues also increased. In addition to improving biochemical parameters, the extract reduced the expression of diabetes-associated genes such as the gene for glucose-6-phosphatase and tumor necrosis factor-*α*, while enhancing the expression of genes involved in glucose transport and metabolism. These findings suggest that the extract may exert its antidiabetic effects through multiple signaling pathways. Extracts of *Euphorbia hirta* Linn have also shown considerable therapeutic potential. In addition to lowering serum levels of cholesterol, triglycerides, creatinine, urea and alkaline phosphatase, the extracts were found to elevate total protein and HDL levels (112). When using plant extracts, it is important to bear in mind that the presence of other components of the non-alkaloid structure may have a synergistic or antagonistic effect. In this context, the choice of extraction technique and solvents, which can increase the selectivity of the extraction process by increasing the concentration of the desired compounds in the mixture while reducing the concentration of interfering components, is very important (113).

Mushrooms, though less extensively explored for their alkaloid content, do contain alkaloid-like nitrogenous compounds with potential antidiabetic properties. Genera such as *Inocybe*, *Psilocybe*, and *Amanita* produce various indole alkaloids, and preliminary research suggests that some may modulate glucose homeostasis, although further investigation is warranted. Additionally, *Cordyceps sinensis* contains cordycepin (3′-deoxyadenosine), an adenosine analog with reported antihyperglycemic and insulin-sensitizing effects (96).

However, it is crucial to consider that the efficacy of alkaloid-containing extracts depends on the presence of other bioactive constituents, which may have synergistic or antagonistic interactions. In this context, the choice of extraction technique and solvents, which can increase the selectivity of the extraction process by increasing the concentration of the desired components in the mixture while reducing the concentration of interfering components, is very important (113).

### 4.4 Polysaccharides from plants and mushrooms in antidiabetic therapy: biosynthesis, mechanisms and therapeutic potential

Polysaccharides derived from both plants and mushrooms represent a broad class of macromolecules with remarkable therapeutic effects, especially in the regulation of blood glucose levels and T2DM management.

These natural polymers exert their antidiabetic action through multiple mechanisms involving physical, enzymatic, hormonal, and cellular pathways. In the small intestine, these polysaccharides can inhibit glucose absorption by increasing the viscosity of the gastrointestinal contents, thereby reducing the rate of gastric emptying. This delay slows down food digestion and consequently lowers carbohydrate absorption (114). In addition, mushroom polysaccharides can bind glucose molecules, thereby decreasing the glucose concentration in the small intestine and limiting its absorption (115). In addition, mushroom polysaccharides can stimulate the endocrine pancreas to adapt to changes in insulin demand by replacing dysfunctional or apoptotic *β*-cells. This regenerative process is particularly significant in the early stages of DM, when the pancreatic β-cell mass retains a substantial capacity for regeneration. The proliferation mechanism of functional β-cells is associated with the expression of the chemokine protein CXCL12 through the activation of the serine/threonine-specific Akt protein kinase, which is part of a prosurvival pathway (116).

In addition, mushroom polysaccharides have been shown to enhance insulin signaling pathways by activating insulin receptors and the PI3K/Akt pathway. Namely, insulin released immediately after a meal triggers the IRS/PI3K/Akt signaling pathway, which regulates lipid and glucose metabolism by promoting lipid deposition and insulin production. In patients with T2DM, this pathway is impaired across various tissues, contributing to insulin resistance and metabolic dysregulation (117).

Similarly, plant-derived polysaccharides, including inulin, pectins, gums, and resistant starches, exert significant effects on glycemic control through similar mechanisms. These soluble fibers form viscous gels in the digestive tract, which slow the absorption of glucose, thereby attenuating postprandial glucose increases. Medicinal plants like *Panax ginseng*, *Momordica charantia* (bitter melon), *Opuntia ficus-indica* (prickly pear), *Astragalus membranaceus*, and *Trigonella foenum-graecum* (fenugreek) contain polysaccharides that have shown remarkable promise in modulating blood glucose levels through various pathways, including the inhibition of digestive enzymes (*α*-glucosidase, α-amylase), modulation of gut microbiota, and improvement in insulin secretion and pancreatic health (118, 119). Recent studies have also suggested that the synergistic effects of polysaccharides combined with other bioactive compounds like polyphenols, flavonoids, and alkaloids enhance the overall therapeutic potential in diabetes management. For instance, the combination of polysaccharides from *O. ficus-indica* with polyphenolic compounds has demonstrated improved glycemic control and a reduction in oxidative damage, offering a more holistic approach to diabetes treatment (120).

### 4.5 Proteins and peptides from plants and mushrooms in antidiabetic therapy: biosynthesis, mechanisms and therapeutic potential

Proteins and bioactive peptides derived from plants and mushrooms have emerged as promising candidates in the development of nutraceuticals and functional foods aimed at preventing and managing T2DM. These macromolecules, particularly when released through enzymatic hydrolysis, possess a wide array of biological functions, including inhibition of key carbohydrate-hydrolyzing enzymes, enhancement of insulin sensitivity, modulation of glucose uptake, and attenuation of oxidative stress and inflammation (121).

Antidiabetic peptides are often derived from storage proteins such as albumins, globulins, prolamins, and glutelins—are a primary source of bioactive peptides, particularly from legumes, cereals, and pseudocereals. For instance, peptide hydrolysates from soy (*Glycine max*), chickpea (*Cicer arietinum*), common bean (*Phaseolus vulgaris*), quinoa (*Chenopodium quinoa*), and amaranth (*Amaranthus hypochondriacus*) have demonstrated significant *in vitro* and *in vivo* antidiabetic activities. These peptides often exert their effects through modulation of insulin receptor substrates (IRS-1), PI3K/Akt signaling, and enhancement of GLUT4 translocation, promoting cellular glucose uptake and insulin sensitivity. Additionally, certain plant-derived peptides possess antioxidant and anti-inflammatory activities that indirectly contribute to improved glycemic control by reducing oxidative stress and inflammation – two major contributors to insulin resistance and *β*-cell dysfunction in T2DM (122).

Mushroom-derived proteins and peptides, while less extensively studied, are increasingly recognized for their antidiabetic potential. For example, *Morchella esculenta* protein hydrolysate inhibited both *α*-amylase and α-glucosidase by 34.93 and 30.56%, respectively (123). Lectins, which represent groups of nonimmune and nonenzymatic proteins or glycoproteins with high structural variability, can be isolated from different sources including mushrooms. Isolated from various edible and medicinal mushrooms, including *Agaricus bisporus*, *G. lucidum*, *Pleurotus ostreatus*, and *Cordyceps militaris*, have demonstrated the ability to modulate glycemic response (124, 125). Their antidiabetic activity is attributed to their interaction with glycosylated receptors on pancreatic *β*-cells and Langerhans islets. This binding induces conformational changes in the cell membrane, facilitating insulin exocytosis and enhancing glucagon release where appropriate. These interactions suggest that mushroom lectins may act as insulin secretagogues, mimicking or enhancing physiological pathways of glucose regulation (126, 127). Additionally, to lectins, low-molecular-weight peptides isolated from mushrooms like *Lentinus edodes* and *G. frondosa* also exhibit *α*-glucosidase inhibitory activity, antioxidant capacity, and enhancement of glucose uptake in muscle cells – further reinforcing the therapeutic potential of mushrooms in diabetes management (122).

## 5 Antidiabetic activities of selected plants and their extracts

### 5.1 *Momordica charantia* (bitter melon)

*M. charantia*, commonly known as bitter melon, has long been recognized for its potential therapeutic benefits in managing diabetes (128). Numerous studies have highlighted its role in regulating blood glucose levels through multiple bioactive compounds, making it a subject of extensive research in the context of antidiabetic therapy. The plant’s fruits, in particular, are rich in various compounds, including charantin, a steroidal saponin, polypeptide-p, an insulin-like peptide, and cucurbitane-type triterpenoids. These bioactive constituents work synergistically to exert hypoglycaemic effects, acting through several mechanisms that target key aspects of glucose metabolism and insulin signaling.

Charantin, one of the key active constituents of bitter melon, is often credited with its insulin-like effects. It has been shown to stimulate insulin secretion from pancreatic β-cells, thereby improving insulin availability in the bloodstream. Additionally, polypeptide-p, another important bioactive molecule from bitter melon, mimics the action of insulin by promoting glucose uptake in peripheral tissues. This insulin-like action helps improve peripheral insulin sensitivity, further enhancing the overall glucose-lowering effect of the plant. Moreover, the cucurbitane-type triterpenoids present in bitter melon have been shown to suppress hepatic gluconeogenesis, a process that contributes to elevated blood glucose levels, particularly in individuals with insulin resistance or T2DM (129, 130).

Beyond its ability to influence glucose metabolism directly, bitter melon also exerts antioxidant effects, which may contribute to its antidiabetic properties. By scavenging free radicals and reducing oxidative stress, bitter melon helps mitigate the cellular damage often associated with chronic hyperglycemia. This antioxidative activity is believed to enhance the plant’s ability to improve insulin sensitivity, a crucial factor in the management of diabetes. Studies have also demonstrated the plant’s capacity to modulate key enzymes involved in carbohydrate metabolism. For example, bitter melon has been shown to increase the activity of hexokinase, an enzyme that catalyzes the phosphorylation of glucose, facilitating its entry into cells and reducing blood glucose levels (131). Conversely, bitter melon inhibits glucose-6-phosphatase, an enzyme involved in the final steps of gluconeogenesis, which further contributes to the reduction of hepatic glucose production (132).

In addition to these inherent mechanisms, extracts from bitter melon have also been studied for their therapeutic potential. Various preparations, including aqueous and ethanol extracts of bitter melon, have demonstrated significant antidiabetic effects in both *in vitro* and *in vivo* studies. These extracts not only retain the hypoglycaemic properties of the whole fruit but also provide a concentrated form of its bioactive compounds, making them more potent in terms of glucose-lowering effects. For instance, bitter melon extracts have been shown to lower blood glucose levels and improve insulin sensitivity in animal models of diabetes. Clinical trials investigating bitter melon extracts have also reported positive outcomes, with some showing reductions in HbA1c levels, a key marker of long-term blood glucose control (133).

One of the advantages of using bitter melon extracts over whole fruit is the ability to standardize the concentration of active compounds, thereby ensuring more consistent therapeutic effects. This standardization also allows for the potential development of bitter melon-based formulations for diabetes management, offering a natural alternative to conventional pharmaceutical treatments. However, despite promising preclinical and clinical findings, the use of bitter melon extracts in diabetes therapy is not without challenges. Variations in the composition of active compounds across different plant varieties and extraction methods can influence the effectiveness of the extracts, and further research is needed to optimize extraction techniques and dosage regimens for maximum therapeutic benefit (134). However, while more clinical studies are needed to establish optimal dosages and long-term safety, bitter melon remains a promising candidate for complementary or adjunctive therapy in the treatment of diabetes, particularly given its role in enhancing insulin sensitivity and regulating blood glucose levels.

### 5.2 *Trigonella foenum-graecum* (fenugreek)

Among medicinal plants with proven antidiabetic efficacy, *T. foenum-graecum* (fenugreek) stands out due to its complex composition and multifaceted biological effects. Traditionally used in Ayurvedic and Middle Eastern medicine, fenugreek seeds are rich in soluble dietary fibers, notably galactomannan, and contain a variety of bioactive molecules, including the unique amino acid derivative 4-hydroxyisoleucine, the alkaloid trigonelline, and a distinct class of steroidal saponins like diosgenin, all of which contribute synergistically to its antidiabetic activity (135).

These compounds exert antihyperglycemic effects through several complementary mechanisms. A particularly important mechanism underlying the antidiabetic potential of fenugreek is its ability to modulate insulin dynamics. The high galactomannan content slows gastric emptying and reduces intestinal glucose absorption, thereby mitigating postprandial blood glucose spikes (135, 136). Meanwhile, 4-hydroxyisoleucine enhances glucose-stimulated insulin secretion from pancreatic *β*-cells without affecting basal insulin levels, by promoting the translocation of the GLUT4 to cell membranes, 4-hydroxyisoleucine also facilitates glucose uptake into muscle and adipose tissues, promoting more physiological insulin release patterns and reducing the risk of hypoglycemia (137). This targeted insulinotropic activity is particularly valuable in early-stage T2DM management, where β-cell function remains partially preserved.

Among the bioactive constituents of fenugreek, trigonelline stands out for its diverse antidiabetic properties. This alkaloid enhances peripheral insulin sensitivity by modulating key signaling pathways, particularly through the upregulation of IRS proteins and activation of the PI3K/Akt cascade. This mechanism facilitates glucose uptake in skeletal muscle and adipose tissue (138, 139). Beyond its role in improving insulin action, trigonelline plays a significant part in glycemic control by regulating hepatic glucose metabolism. Preclinical studies have demonstrated that trigonelline can inhibit critical gluconeogenic enzymes, such as glucose-6-phosphatase and phosphoenolpyruvate carboxykinase, leading to reduced endogenous glucose production (140). In addition to its insulinotropic effects, fenugreek also impacts carbohydrate digestion and absorption. Fenugreek is rich in soluble dietary fiber, particularly galactomannan, which forms a viscous gel in the gastrointestinal tract. This mucilaginous matrix delays gastric emptying, slows enzymatic digestion of carbohydrates, and reduces the rate of intestinal glucose absorption, thereby mitigating postprandial glucose spikes. Furthermore, fenugreek seed extracts and isolated compounds inhibit digestive enzymes such as *α*-amylase and α-glucosidase, further decreasing carbohydrate hydrolysis and absorption. Additionally, trigonelline exhibits notable antioxidant properties, helping to mitigate oxidative stress and protect pancreatic *β*-cells, which are particularly vulnerable to damage in the hyperglycemic environment of diabetes (141). Several bioactive compounds in fenugreek, including polyphenols and saponins, demonstrate strong free radical scavenging activity, further protecting pancreatic β-cells from oxidative stress-induced damage, a critical factor in the pathogenesis and progression of both T1DM and T2DM (142).

Extracts of fenugreek seeds, prepared through aqueous, alcoholic, or hydroalcoholic methods, retain a broad spectrum of bioactive compounds and have been extensively evaluated in diabetic models and clinical trials (143). Clinical interventions have consistently demonstrated that supplementation with fenugreek seed powder or extracts leads to significant reductions in fasting plasma glucose, postprandial blood glucose levels, and HbA1c, often accompanied by improvements in lipid profiles, including reductions in total cholesterol, LDL cholesterol, and triglycerides (144, 145). These multifactorial benefits are particularly valuable considering the close association between T2DM and dyslipidemia, where managing both glycemic control and lipid metabolism plays a critical role in reducing cardiovascular risk (146). Importantly, fenugreek’s safety profile has been favorable across studies, with minimal adverse effects reported even at relatively high doses (147, 148). Nevertheless, standardization of extracts with respect to key active markers such as 4-hydroxyisoleucine and trigonelline remains a necessary step for optimizing clinical efficacy and ensuring reproducibility across future therapeutic applications (149).

### 5.3 *Cichorium intybus* (chicory)

*Cichorium intybus*, commonly known as chicory, is a perennial herb widely distributed in the Mediterranean and Balkan regions, traditionally used for a variety of medicinal purposes, including the management of diabetes. Its bioactive compounds, particularly inulin, flavonoids, polyphenols, and sesquiterpene lactones, contribute significantly to its antidiabetic effects. Among these, inulin, a soluble dietary fiber found in chicory roots, is of particular importance in modulating blood sugar levels. Unlike digestible carbohydrates, inulin is not absorbed in the small intestine, thereby slowing glucose absorption and helping to reduce postprandial glucose spikes. This action makes chicory an effective plant for controlling blood glucose levels after meals, a critical challenge in managing T2DM (150, 151). Additionally, Birsa and Sarbu (151) highlight inulin’s prebiotic effects, which foster the growth of beneficial gut microbiota. This enhancement of gut health not only supports digestion but may also improve insulin sensitivity, further contributing to better metabolic outcomes.

In addition to inulin, chicory contains a variety of other bioactive compounds that play an essential role in regulating glucose and lipid metabolism. These compounds exhibit a combination of antioxidant, anti-inflammatory, and insulin-sensitizing properties, all of which are crucial for mitigating the complications associated with diabetes. One key bioactive constituent, triterpenoids, has been shown to enhance insulin sensitivity by improving insulin receptor activity, which facilitates glucose uptake in peripheral tissues such as muscle and adipose tissue (152). Chicory’s effects are not limited to improving glucose metabolism, it also contributes to liver health. The hepatoprotective properties of chicory root extract have been highlighted in several studies, where it was shown to reduce liver damage associated with non-alcoholic fatty liver disease, a condition often linked to insulin resistance in diabetes. By protecting the liver, chicory may help to mitigate some of the systemic complications often observed in diabetes (153). The work of Khan and Chandra (152) underscores that these compounds can effectively mitigate oxidative stress and inflammation, two critical factors in the progression of diabetes. The antioxidant capacity of chicory is particularly noteworthy; it may protect pancreatic *β*-cells from oxidative damage, thereby preserving their function and promoting insulin secretion. This is crucial, as chronic hyperglycemia can lead to β-cell dysfunction, exacerbating the challenges associated with insulin insufficiency.

Moreover, chicory’s hepatoprotective effects are significant, particularly in the context of diabetes management. Kt and Sivalingam (154) provide evidence that chicory extract can mitigate streptozotocin-induced damage to pancreatic β-cells by inhibiting NF-κB activation and reducing oxidative stress. This protective action is essential for maintaining healthy pancreatic function, which is vital for effective glucose metabolism and overall metabolic health. Chicory also offers cardiovascular benefits, which are particularly relevant for individuals managing diabetes. Ebrahiminia et al. (155) report that chicory root extract significantly lowers fasting blood glucose levels and HbA1c in patients with T2DM, highlighting its efficacy as a therapeutic agent. Krepkova et al. (156) further support these findings by demonstrating improvements in lipid profiles, including reductions in total cholesterol and triglycerides, which are crucial for mitigating cardiovascular risks often associated with diabetes.

Chicory extracts are typically prepared through aqueous, alcoholic, or hydroalcoholic extraction methods. These extracts retain a high concentration of inulin and other bioactive compounds, which are critical for their therapeutic benefits. However, the method of extraction used for chicory is another critical factor influencing the efficacy of its bioactive compounds. Singh et al. (157) emphasize the importance of optimizing extraction techniques to maximize the yield of inulin and other beneficial compounds. Their findings suggest that both aqueous and hydroalcoholic extraction methods can produce effective extracts, enhancing chicory’s potential as a functional food and nutraceutical. Research has shown that chicory extracts can maintain their efficacy in reducing blood glucose levels, improving insulin sensitivity, and lowering lipid levels when administered either in isolation or as part of a combined treatment approach (158). The ease of obtaining chicory extract makes it an attractive option for developing functional foods or nutraceuticals aimed at diabetes management.

Importantly, chicory has a favorable safety profile, with minimal adverse effects reported, even at relatively high doses. This makes it a promising natural therapeutic option for long-term diabetes management. However, the necessity for standardization of chicory extracts to ensure consistent potency and effectiveness. This standardization is critical for clinical applications and for maximizing the therapeutic benefits for individuals with diabetes (158).

Further selected medicinal plants with antidiabetic properties, bioactive components, effective doses and mode of action are listed in Table 2.

**Table 2:** Activity of medicinal plants with antidiabetic properties.

Plant species	Extract	Bioactive components	Model	Activity	Effective dose	Reference
*Ammodaucus leucotrichus*	Water infusion of fruits	Phenolic compounds	*In vivo*	Inhibition of AR and antidiabetic effect in alloxan-induced diabetic rats	150–300 mg/kg	(201)
*Cichorium intybus*	Soxhlet extracts (80% methanol) of roots	Pelargonidin, Magnolialide, Apigenin, Jacquilenin and Kaempferol	*In*-*silico* and *in vitro*	Inhibitory effects against AR enzyme	IC_50_ = 15.37 ± 3.41 μg/mL	(202)
*Glaucosciadum cordifolium*	Areal parts (96% ethanol)	Phenolic compounds	*In vitro*	Inhibition of amylase and glucosidase	Amylase inhibition 0.34–0.71 mmol ACAE/g Glucosidase inhibition 0.64–0.71 mmol ACAE/g	(198)
*Corydalis* species (*C. solida; C. erdelii*)	Ethanol extracts, and water infusion of areal parts	Phenolic compounds	*In vitro*	Inhibition of amylase and glucosidase	*C. solida*: 0.39 (water) and 0.05 mmol ACAE/G (ethanol) for amylase; 0.99 mmol ACAE/g of ethanolic extracts for glucosidase *C. erdelii*: 0.43 (water) and 0.05 mmol ACAE/g of (ethanol) for amylase; 0.87 mmol ACAE/g of ethanolic extracts for glucosidase	(199)
*Coleus forskohlii*	Water extracts of fresh leaves	Phenolic components; Rosmarinic acid	*In vitro*	Inhibition of human recombinant ALR2	IC_50_ = 20 μM	(203)
*Helichrysum italicum*	Essential oils isolated from fresh, naturally dried, and freeze-dried flowers	Sesquiterpene hydrocarbons, monoterpene hydrocarbons	*In vitro*	Inhibition of amylase and glucosidase	0.19–2.01 mmol ACAE/g	(204)
*Matricaria chamomilla* L.	Essential oils of dried flower heads	Oxygenated sesquiterpenes (bisabolol oxides A and B) Non-terpene compounds (spiroethers)	*In vitro*	Inhibition of amylase and glucosidase	1.51–1.54 mmol ACAE/g	(72)
*Opopanax hispidus*	Areal parts (96% ethanol)	Phenolic compounds	*In vitro*	Inhibition of amylase and glucosidase	Amylase inhibition 0.35–0.46 mmol ACAE/g Glucosidase inhibition 1.67–1.77 mmol ACAE/g	(181)
*Rosa abyssinica*	70% Ethanolic Fruit	Alkaloids, Phenolic compounds	*In vitro* and *in vivo*	Reduction of postprandial hyperglycemia through α-amylase inhibition *in vitro* and lower BGL *in vivo* in both glucose-loaded and STZ-induced diabetic mice with minimal risk of hypoglycemia	α-amylase inhibition IC_50_ = 21.37 ± 4.252 μg/mL	(205)
*Typha domingensis* Pers	*n*-Butanolic fraction of whole plant methanol extract	Tannins, Polyphenols, Fattyacids, Sesquiterpenes, Phenolic acids, Sugar alcohols, Amino acids, Glycosides, Tropane alkaloids, Phenolic glycosides, Terpenoids, Sphingosines, Flavonoids, and Glycerolipids.	*In vitro, in-silico*, and *in-vivo*	The molecular docking showed better binding affinities for α-amylase and α-glucosidase. The ADMET analysis confirmed its potential in antidiabetic activity.	α-amylase inhibition IC_50_ = 8.42 ± 0.21 μg/mL α-glucosidase inhibition IC_50_ = 1.89 ± 0.14 μg/mL	(206)
*Taraxacum officinale*	70% ethyl alcohol root extract (1:10 g/mL)	Inulin, Phenolic acids, Sugar alcohols, Amino acids, Glycosides,	*In vitro*	Inhibition of amylase.	IC_50_ = 106.67–450.11 μg/mL depending on the variety	(207)
*Withania frutescens*	70% ethyl alcohol extract of leves (1:10 g/mL)	Terpinene-4-ol, Ferrocene, Phenazine	*In vitro, in vivo*	The continuous treatment of diabetic mice with the extract of *Withania frutescens* for 4 weeks succeeded to slowly manage their high fasting blood glucose levels (after 2 weeks). The *in vitro* assays demonstrated that the inhibition of alpha-amylase and alpha-glucosidase.	400 mg/kg	(208)
*Vaccinium myrtillus*	Water and 80% ethyl alcohol extract of leaves with stem parts (1:10 g/mL)	Quercetin and Kaemferol derivatives, Chlorogenic and *p*-coumaric acid.	*In vitro*	Inhibition of glucosidase	IC_50_ = 0.29 ± 0.02 mg/mL	(209)
*Polygonum maritimum*	Methanol and dichloromethane (1:40, w/v)	β-sitosterol, Stigmasterol, 1-octacosanol and Linolenic acid	*In vitro*	Inhibition of α-amylase and α-glucosidase	IC_50_ = 2,527 μg/mL	(210)
*Ximenia americana extracts*	Extract of leaves obtained bz different solvents	9,12-Octadecandionoic acid	*In vitro*	Inhibition of α-amylase	IC_50_ = 84.58–177.08 μg/mL	(211)
*Annona muricata* Linn.	70% ethyl alcohol (1:5 g/mL) of pant leaves	Chlorogenic acid, Procyanidin B2 and C1, (epi)catechin, Quercetin-diglucoside, Quercetin-glucosyl-pentoside and Rutin	*In vitro*	Inhibition of α-amylase and α-glucosidase, pancreatic lipase, glycation	Inhibition of glycation in BSA-fructose (IC_50_ = 45.7 ± 13.5 μg/mL), α-amylase inhibition (IC_50_ = 9.2 ± 2.3 μg/mL), α-glucosidase (IC_50_ = 413.1 ± 121.1 μg/mL) and lipase (IC_50_ = 74.2 ± 30.1 μg/mL).	(212)
*Prosopis cineraria*	Chloroform fraction of 70% methanolic extracts of stem barks	Methyl 5-tridecyloctadec-4-enoate and Nonacosan-8-one, Lupeol, β-sitosterol and Stigmasterol	*In vitro, in vivo*	Treatment significantly lowered the level of blood glucose, glycosylated hemoglobin and also restored body weight, liver glycogen content and serum insulin level in diabetic rats. Extract also showed significant inhibition of α-amylase enzyme activity	α-amylase inhibition IC_50_ = 40.29 μg/mL.	(213)
*Physalis alkekengi*	75% ethyl acetate extracts of aerial parts of plant and fruit (7:3) 75% ethyl acetate extracts of aerial parts of plant and fruit (7:3)	Flavonoids, Physalins and Phenolic acids	*In vitro, in vivo*	*In vivo*, extract significantly decreased the levels of fasting blood glucose and fasting insulin, as well as total cholesterol and triglyceride, in the pre-diabetic rats. The *in vitro* effects included reduction of oxidative stress, inhibition of α-glucosidase, and enhancement of insulin sensitivity with reduced CYP2E1 expression and enhanced GLUT4 expression/function	α-glucosidase inhibition IC_50_ = 2.90 μg/mL (for aerial parts) and 4.04 μg/mL (for fruits)	(214)
*Abelmoschus esculentus*	Methanolic fruit extract of	Phenols and Flavonoids	*In silico*, *in vitro*	*In silico* analysis, revealed that ligand molecule could bind with the α-glucosidase and α-amylase molecules. Extract significantly reduced α-amylase and α-glucosidase activities compared to the most common drug.	α-amylase inhibition = 14.36–19.23% at 50–200 μg/mL α-glucosidase inhibition = 15.89–37.19% at 50–200 μg/mL	(215)
*Zanthoxylum alatum*	Ethanolic extract of whole dried plant	Polyphenolic, flavonoids, terpenoids, anthraquinones and saponins type of compound	*In vitro, in vivo*	*Zanthoxylum alatum extract* potential to inhibit PTP-1B activity and to stimulate the glucose uptake by C2C12 myotubes exhibited significant lowering of blood sugar in STZ-induced Sprague–Dawley rats.	NA	(187)

NA, not applicable.

## 6 Antidiabetic activities of selected mushrooms and their extracts

### 6.1 *Morchella* spp.

*M. esculenta* has been traditionally cultivated in China, Malaysia and Japan for over 2,000 years (159, 160). In traditional medicine, it is recognized for its ability to improve digestion, alleviate inflammation and promote overall well-being. The functional properties of this mushroom are attributed to its diverse bioactive compounds, including polysaccharides, proteins, peptides, amino acids, terpenes, lipids and essential minerals, similar to other medicinal mushroom species (159, 161). In the study by Guo et al. (159) the antidiabetic potential of bioactive polysaccharides from mycelia and broth of submerged fermentation was evaluated. Intracellular polysaccharides (IPS) were extracted from mycelial powder using hot water (90°C, 2 h), while extracellular polysaccharides (EPS) were obtained from concentrated fermentation filtrate. Polysaccharides were further deproteinized, dialyzed and lyophilized. Both IPS and EPS showed dose-dependent inhibitory effects against *α*-amylase and α-glucosidase (from 0.5 to 2.5 mg/mL), likely due to their triple-helix structures which allow the active sites and fragments to enhance functionality. EPS exhibited a stronger inhibitory effect of *α*-amylase and α-glucosidase (21.59 and 6.64%, respectively) than IPS. This may be attributed to its lower molecular weight (MW) – 109 kDa, which facilitates cellular uptake. However, too low MW of polysaccharides may reduce biological activity by limiting structural complexity. In addition, EPS with higher galactose and sulfate content correlated linearly with enzyme activity and a decrease in serum glucose levels (159). *In vitro* salivary, gastric and intestinal digestion of *M. esculenta* exopolysaccharide enhanced *α*-amylase inhibitory activity. The exopolysaccharides were partially degraded leading to changes in chemical composition, MW and structural characteristics (161). Hot water-extracted and deproteinized polysaccharide extract from *M. esculenta* fruiting bodies (MEP) showed an effect on lowering fasting blood glucose levels and improving glucose tolerance in diabetic mice induced by STZ and high-fat diet. MEP lowered proinflammatory cytokines (IL-1β, TNF-α and IL-6), which contribute to insulin resistance, indicating its potential to regulate hyperglycemia and hyperlipidemia. Additionally, MEP modulated the gut microbiota by decreasing harmful taxa such as *Acinetobacter* and increasing Firmicutes, *Lactobacillus*, which are important for the hypolipidemic effect, and *Prevotella*, which is related to glucose metabolism (160). Shurong et al. (162) also investigated similar effects using *Morchella importuna* polysaccharides (MIP) *in vitro*. MIP exhibited excellent water and oil holding capacity, emulsifying ability, foaming and rheological properties. Gastrointestinal digestion reduced its MW significantly. Since polysaccharides cannot be fully digested in the stomach, they can be utilized by microorganisms in the intestine that produce short-chain fatty acids (SCFAs). Post-fermentation SCFA levels rose from 3.23 mmol/L to 39.12 mmol/L, with increased propionic acid content highlighting the potential of fungal polysaccharides to reshape the intestinal microbiota. Although MIP did not improve the richness and diversity of the gut microflora, it increased the relative abundance of Firmicutes, while Bacteroidetes and Proteobacteria were reduced. Previous research has confirmed that tight junctions and mucins play a critical role in maintaining the integrity of the intestinal barrier. Hyperglycemia can alter the tight junctions and adhesion junctions, leading to intestinal leakage, causing exogenous substances to enter the bloodstream and damage pancreatic *β*-cells, which is a pathway in the pathogenesis of diabetes. Firmicutes can promote tight junction bending and the synthesis of mucin, while *Bacteroides* exert the opposite role (163). An *in vivo* study using methanolic extract of *Morchella conica* demonstrated a significant reduction in blood glucose levels in diabetic mice from 346 mg/dL to 132 mg/dL following treatment with extract in concentration of 100 mg/kg body weight. *In vitro*, the extract inhibited 80% of protein tyrosine phosphatase 1B (PTP1B) activity, with a lower IC_50_ value (26.5 μg/mL) compared to oleanolic acid as a positive control (IC_50_ 36.2 μg/mL) (164). PTP1B, is a key negative regulator of insulin signaling and its inhibition can target both diabetes and obesity (165). The *α*-amylase inhibition of the crude methanolic extract of *M. conica* was dose-dependent with a low IC_50_ value (77.74 ± 0.018 μg/mL). Extract expressed high antioxidant potential and antiglycation test showed a positive correlation between the concentration of the extract and its activity, with a low IC_50_ value (24 μg/mL). Furthermore, histological analysis showed that the extract protected kidney, liver and pancreatic tissues from damage (164).

### 6.2 Hericium erinaceus

*Hericium erinaceus* is a medicinal mushroom rich in bioactive compounds including (polysaccharides, glycoproteins, phenolic compounds, alkaloids, sterols, fatty acids, laccase, lectones, etc.) which exhibit a wide range of pharmacological functions (39, 49). *β*-glucans extracted from the *H. erinaceus* using sodium hydroxide and the enzymes β-1,3-glucanase and β-1,6-glucanase demonstrated an inhibitory effect on wheat starch digestion. High MW β-glucans and triple helix structure showed greater ability to bind hydrolyzed starch fragments and thus to more effectively inhibit starch digestion. In addition, β-1,3-glucan showed stronger inhibition than β-1,6-glucan due to better cross-linking capability with starch. Lower starch digestibility contributes to a lower glycemic index and prevention of diabetes (39). Another polysaccharide, HEP-1, from the fruiting body of *H. erinaceus* with low MW, showed promising effects in a T2DM model by improving glucose and lipid metabolism. It increased serum glucose uptake through hepatic glycogen synthesis via activation of the IRS/PI3K/AKT signaling pathway. It also suppressed fatty acid synthesis and hepatic lipid accumulation through the AMPK/SREBP-1c signaling pathway. In addition, HEP-1 positively influenced gut microbiota diversity and richness by increasing the abundance of *Dubosiella*, *Akkermansia* and *Lactobacillus*, and reducing *Bacteroides* and Rikenellaceae RC9. Higher levels of *Dubosiella* and *Lactobacillus* were associated with a lower risk of liver damage. Beneficial metabolites were increased in the liver via the gut-liver axis, preventing the occurrence of T2DM (166).

Chromatographic separation of the n-hexane soluble fraction of *H. erinaceus* revealed ten active compounds with *α*-glucosidase activity, including four novel compounds (erinaceol A, B, C and D). Among them, erinaceol D and other already known compounds: 4-[3′,7′-dimethyl-2′,6′-octadienyl]-2-formyl-3-hydroxy-5-methyoxybenzyl alcohol, hericene A, hericene D and hericenone D showed potent α-glucosidase inhibition (IC_50_ < 20 μM), surpassing the activity of acarbose. Structural analysis indicated that long polar side chains of hericene A, hericene D and hericenone D, while for ericenol D and 4-[3′,7′-dimethyl-2′,6′-octadienyl]-2-formyl-3-hydroxy-5-methyoxybenzyl alcohol were the major contributors to activity (167). *In vivo* data showed that administration of *H. erinaceus* mushroom aqueous extract (AEHE) to STZ-induced diabetic rats reduced blood glucose levels and increased insulin levels in a dose-dependent manner. Consequently, improved glycemic control prevented body weight loss. That is caused by the excessive breakdown of muscle structural proteins into amino acids used for gluconeogenesis in insulin-deficient states. In addition, AEHE enhanced hepatic antioxidant enzyme activities (SOD, CAT and GSH-Px), and decreased lipid peroxidation (49).

### 6.3 *Coriolus versicolor* (*Trametes versicolor* (L.: Fr.) Lloyd, 1920)

*Coriolus versicolor* or *Trametes versicolor* is a medicinal mushroom recognized as a rich source of biologically active polysaccharides and polysaccharopeptides. Commercial preparations such as Krestin (PSK) and polysaccharopeptide (PSP) are produced by submerged mycelium cultivation and extraction (168).

Su et al. (169) examined the inhibitory effects of n-hexane extracts from six mushroom fruiting body powders: *G. frondosa*, *H. erinaceus*, *Agaricus blazei*, *G. lucidum*, *C. versicolor* and *Phellinus linteus* on digestive enzymes *α*-amylase and α-glucosidase. *C. versicolor* extract showed the strongest α-amylase inhibition (IC_50_ = 1.20 mg/mL), while the extract of *A. blazei* had the weakest effect (IC_50_ = 6.90 mg/mL). Even though the activity of acarbose (IC_50_ = 0.039 mg/mL) was stronger, all tested n-hexane extracts showed stronger *α*-glucosidase inhibition (IC_50_ = 0.0376–0.165 mg/mL) than acarbose (IC_50_ = 4.69 mg/mL). The presence of unsaturated fatty acids, particularly oleic and linoleic acid, appeared to contribute significantly to α-glucosidase inhibition. When oleic acid and linoleic acid were tested individually, both exhibited higher activity IC_50_ = 0.0220 mg/mL and IC_50_ = 0.0327 mg/mL, respectively than acarbose (IC_50_ = 4.69 mg/mL), but showed lower inhibitory effect on α-amylase. Crude soybean and sunflower oils, rich in these acids possessed weak α-amylase and moderate α-glucosidase inhibitory effects. Extracellular polysaccharopeptides (ePSP) from *T. versicolor* LH-1 strain were investigated for their ability to regulate glucose homeostasis in insulin-resistant human hepatoma HepG2 cells. At concentrations below 2000 μg/L, TV LH-1 ePSP was not toxic. In the normal glucose (NG) model, it enhanced glucose uptake by 30 and 50% at 500 μg/mL and 1,000 μg/mL, respectively, compared to 12% increase by 1,000 μg/L PSK. In high glucose (HG) or high glucose, high insulin (HGI) models, TV LH-1 ePSP (500 and 1,000 μg/mL) significantly increased glucose uptake, by 20 to 50%, outperforming PSK (1,000 μg/mL) which increased glucose uptake by approximately 20%. Similar results were also obtained in the HGI model. The results confirmed that TV LH-1 ePSP increased glucose uptake in a dose-dependent manner in all three models (170). TV-ePSP was administered orally (0.1, 0.5 or 1.0 g/kg per day) to male Wistar rats with T2DM that was induced by high-fat diet and STZ (171). It attenuated the increase in blood glucose levels, significantly reduced oxidative stress and increased SOD activity in plasma as well as increased glutathione levels in plasma and erythrocytes. These findings suggest that TV-ePSP possesses both antihyperglycemic and antihypertriglyceridemic properties (172). Xian et al. (173) investigated the potential application of *C. versicolor* water extract with high polysaccharide content (74.17% (w/w)) obtained from a commercially available mushroom in the treatment of DM. The extract was tested for its antidiabetic and anti-insulin-resistant effects in myoblasts (L6 cells) and skeletal muscles of rats with T2DM. Rats received daily intragastric doses of 25, 50 and 100 mg/kg and were compared with STZ alone and STZ plus Met (100 mg/kg/day). After four weeks, the *C. versicolor* extract showed similar or even stronger blood glucose-lowering effect than Met. Increase of extract dose from 25 to 100 mg/kg did not significantly enhanced efficacy, suggesting high potency at lower doses. Moreover, the result of the 25 mg/kg/day administration was similar to that of 100 mg/kg/day Met administration, indicating a higher efficacy of the *C. versicolor* extract. In addition, glucose consumption in insulin-resistant L6 cells decreased compared to the control group, while it increased after treatment with Met (1 mM, 24 h) and *C. versicolor* extract (100 mg/mL and 200 mg/mL, 24 h), suggesting concentration-dependent manner under insulin-resistant conditions.

In rats with DM, cardiac function was significantly reduced, as indicated by left ventricular ejection fraction (EF%) and fractional shortening (FS%) to 52.88 ± 1.23% and 26.87 ± 1.06%, respectively, compared to the sham group. Cardiac function was improved with *C. versicolor* extract (25 mg/kg/day), restoring EF% and FS% to 71.99 ± 1.03% and 42.79 ± 0.87%, respectively. Histological analysis revealed that *C. versicolor* extracts reduced structural abnormalities and interstitial fibrosis, suggesting that *C. versicolor* extract has the potential to alleviate cardiac dysfunction (174).

The therapeutic effect of *C. versicolor* mushroom fruiting body powder on STZ-induced diabetic mice using different doses (1,000 mg/kg – LD, low dose; 2000 mg/kg -MD, medium dose; 4,000 mg/kg – HD, high dose) was investigated (175). Normal control group (NS) and the diabetic control group (DC) received normal saline, while the positive control group (PC) received 200 mg/kg Met. After 28 days, fasting blood glucose concentration in *C. versicolor*-treated mice decreased to the initial concentration. Oral glucose tolerance test showed significantly reduced blood glucose levels in both the PC and HD groups compared to the DC group (*p* < 0.05). The results indicate that *C. versicolor* can protect pancreatic *β*-cells and improve insulin resistance, accelerate the breakdown and utilization of glucose, and accelerate glucose metabolism.

To investigate the protective effect of *T. versicolor* crude extract (TVP) on bone health in diabetes, Wistar rats were divided into control or a high-fat diet groups. Diabetic rats received either distilled water (vehicle group-VH) or TVP at 0.1 g/kg weight. Compared to the VH group, subjects had significantly lower postprandial blood glucose levels, reduced femoral cortical porosity, increased tibial bone volume and femoral bone strength, and less DM-induced deterioration of proximal tibial microarchitecture. The protective effect of TVP on bone properties was mediated in part by the improvement in hyperglycemic control in the DM animals (176). Additionally, two highly methylated cyclic heptapeptides with proven antimicrobial activity isolated from the *C. versicolor* mushroom- ternantin and [D-Leu7]ternatin, a ternatin derivative-have been shown to have an inhibitory effect on fat accumulation in 3 T3-L1(adipocytes) (177, 178). When administered to KK-Ay diabetic mice via a subcutaneous osmotic pump (8.5 or 17 nmol/day ternatin and 68 nmol/day [D-Leu7]ternatin), these compounds did not affect body or adipose tissue weight, but both suppressed hyperglycemia, suggesting a preventive effect on hyperglycemia and fatty acid synthesis (179).

Further selected mushrooms with antidiabetic properties, bioactive components, effective doses and mode of action are listed in Table 3.

**Table 3:** Activity of mushrooms with antidiabetic properties.

Mushroom species	Extract	Bioactive components	Model	Activity	Effective dose	Reference
*Agaricus blazei* Murill	Etanol extract (EE) and Etyl acetate extract (EA) of fruiting bodies	Phenolic compounds	*In vitro*/HepG2 cells	EA showed stronger antioxidant activity and inhibition of α-glucosidase compared to EE. Both extracts improved glucose uptake by HepG2 cells, with EA showing similar glucose-lowering activity as Met.	At 0.5 to 8 mg/mL α-glucosidase inhibition of EA = 49.82 to 73.45%	(216)
*Coprinus commatus*	Fruiting body ethanol extract	Flavonoids, alkaloids, terpenoids, vitamin C, vitamin E, rutin, saponin	*In vivo*/STZ-induced diabetic rats	Reduced HbA1c, blood glucose and DPP-4 enzyme levels, increased GLP-1, GSH and insulin levels.	250–750 mg/kg	(217)
*Coprinus commatus*	Mycelia water extract from submerged cultivation	Polysaccharides	*In vivo*/STZ-induced diabetic mice model	Improved insulin resistance and energy metabolism, suppressed kidney dysfunction, relieved renal oxidative stress and inflammation, reversed renal injury; Preventions of diabetic nephropathy.	400 mg/kg	(218)
*Floccularia luteovirens*	Deproteinizated hot water extract of fruiting bodies	Polysaccharide extract	*In vivo*/db/db mouse DN model	Improved glucose and lipid metabolism, inhibited excessive weight gain, relieved kidney tissue injury, activated NRF2/HO-1 pathway, and enhanced CAT activity in diabetic nephropathy complications.	100, 200 and 400 mg/kg	(219)
*Fommes fomentarius*	Extracellular polysaccharide	Selenium-polysaccharides	*In vivo*/STZ- induced diabetic rats	Declined blood glucose, improved insulin secretion and body weight and decreased hemoglobin A1c levels, improved lipid profiles and liver, pancreas, and kidney tissues.	150 mg/kg	(220)
*Ganoderma applanatum*	Water and metalnol extract of fruiting bodies	Not specified	*In vivo*/Alloxan induced diabetic rats	Improved body weights; reduction in serum cholesterol, LDL cholesterol and tryglicerides; reduction in blood glucose; reduction in liver marker enzymes	250 and 500 mg/kg	(221)
*Ganoderma lucidum*	Purified hot water extract of fruiting bodies	FYGL-Proteoglycan	*In vivo*/diabetic rats model	Acted on skeletal muscles β-subunit of insulin receptor by increase tyrosine phosphorilation level due to decreased expression of PTP1B in skeletal muscles.	50 and 150 mg/kg	(222)
*Ganoderma lucidum*	Purified hot water extract of fruiting bodies	FYGL-Proteoglycan polysaccharide fraction (82%)	*In vivo*/diabetic mice model	Enhanced antioxidant enzyme activities in liver, i.e., SOD, CAT and GSH-Px by 35.4, 137%, 48,5%, respectively.	75, 250 and 450 mg/kg	(48)
*Ganoderma lucidum*	Purified hot water extract of fruiting bodies	F31 fraction: β-heteropolysaccharide composed of polysaccharides (65.5%), uronic acid (18.7%) and protein (15.1%)	*In vivo*/STZ-induced diabetic mice	Expressed hypoglycemic activity through the decrease of fasting serum glucose and insulin levels, as well as epididymal fat/BW ratio.	25 and 50 mg/kg	(223)
*Heimioporus retisporus*	Purified hot water extract of fruiting bodies	Water soluble neutral heteropolysaccharide	*In vivo*/STZ-induced diabetic mice model	Reduced blood glucose levels, decreased heart organ index and reduced IL-6 and TNF-α expression.	40 mg/kg	(224)
*Hohenbuehelia serotina*	Purified hot water extract of fruiting bodies	Neutral polysaccharide extract	*In vivo*/diabetic mice model	Lower blood glucose level, reduce liver index, might prevent hepatic steatosis or hepatomegaly.	200 mg/kg/day	(225)
*Inonnotus obliqus*	Methanol extract of fruiting bodies	Not specified	*In vitro*	Among six mushroom methanol extracts that were tested, all showed better affinity for α-glucosidase inhibitory activity than α-amylase activity *I. obliqus* expressed the best activity	α-glucosidase activity IC_50_ = 220.31 mg/mL	(226)
*Inonnotus obliqus*	Methanol extract of fruiting bodies	Flavonoids, Coumarin, Tannin, Anthracenes, Terpenoids and Glycosides, Endogenous Metabolites, Steroids, Fatty Acyls, Carboxylic acids	*In vivo*/C57BKS-db mice and C57/BKS mice	Decrease body weight and fasting blood glucose levels while mitigating the severity of lesions in the intestines, liver, kidneys, and pancreas of diabetic mice. Modified the composition of intestinal flora in db/db mice and ameliorated intestinal microecological disorders.	600 mg/kg	(226)
*Lentinus edodes*	Ethanol extract (EE) and Hexan extract (HE) of fruiting bodies	Glucans and Proteins	*In vitro*	HE expressed stronger α-amylase activity and α-glucosidase activity than EE.	HE α-amylase activity IC_50_ = 127 mg/mL; α-glucosidase activity = 12.9 mg/mL	(227)
*Lentinus edodes*	Purified hot water extract of fruiting bodies	Polysaccharide	*In vivo*/STZ, high fat and high sugar diet induced type 2 diabetic mice	Regulate the oxidative stress response; Influenced on physiological and biochemical indexes and histopathological changes.	50, 100 and 200 mg/kg	(228)
*Lentinus edodes and Hypsizigus marmoreus*	Mushrooms’ fruiting bodies powder	Agmatine, Sphingosine, Pyridoxine, Linolenic and Alanine	*In vivo*/Alloxan-induced diabetic rats	Lower plasma glucose levels and modulate intestinal microbiota in diabetic individuals.	25% of mushroom powder + 75% of commercial diet	(229)
*Phaeogyroporus portentosus*	Ethanol extract (EE) and Hexan extract (HE) of fruiting bodies	Glucans and Proteins	*In vitro*	HE expressed stronger α-amylase activity while EE had stronger α -glucosidase activity than EE.	HE α-amylase activity: IC_50_ = 15.7 mg/mL EE α-glucosidase activity: IC_50_ = 203 mg/mL	(227)
*Pleurotus cornucopiae*	Aqueous extract	Saponins, Flavonoids, Tannins	*In vivo*/alloxan induced diabetes in male Wistar rats	Lowered blood glucose level, CAT, and GSHPx activities; increased SOD and Nrf2 activity.	500 mg/kg	(230)
*Pleurotus ostreatus*	Hexan extract (HE) and Methanol extract (ME)	Non specified	*In vitro*	HE expressed the strongest inhibitory activity on α-glucosidase among the twenty-four mushrooms tested	HE α-glucosidase activity: IC_50_ = 0.10 mg/mL ME α-glucosidase activity: IC_50_ > 15.7 mg/mL	(37)
*Phellinus fastuosus* *Phellinus sanfordii*	Crude hydroalcoholic (70% ethanol) extract	Non specified	*In vitro* *In vivo/*oral starch tolerance and oral glucose tolerance test – Male Wistar albino rats	Among six tested mushroom extracts, the extracts of *P. fastuosus* and *P. sanfordii* exhibited the best antihyperlipidemic potential *in vitro* by inhibiting α-amylase and α-glucosidase activity; Improved tolerance of rats to starch and glucose was dose-dependent The blood glucose concentration decrease was greater in starch-fed compared to glucose-fed rats.	IC_50_ (mg/mL) for *in vitro* starch digestion assay: *P. fastuosus* 27.33 *P. sanfordii* 30.33 200 and 400 mg/kg	(231)
*Pholiota adiposa*	Purified ethanol extract of fruiting bodies	Ergosta-4, 6, 8(14), 22-tetraen-3-one (ETO)	*In vivo*/ STZ- induced diabetic mice model	Decreased fasting blood glucose level, tryglycerols, total and low-density lipoprotein cholesterol and increased the levels of SOD, CAT, GSH-Px; restored pancreatic islet morphology and function.	0.1 and 0.05 mmol/kg/day	(232)
*Schizophyllum commune*	Ethanol extract (EE) and Hexan extract (HE) of fruiting bodies	Glucans and Proteins	*In vitro*	HE expressed stronger α -amylase activity while EE had stronger α -glucosidase activity than EE.	HE α -amylase activity: IC_50_ = 15.7 mg/mL EE α-glucosidase activity: = 50.9 mg/mL	(227)
*Suillus granulatus*	Hexan extract (HE) Methanol extract (ME)	Not specified	*In vitro*	HE expressed the strongest α-amylase inhibitory activity among the twenty-four mushrooms tested	HE α -amylase activity: IC_50_ = 0.13 mg/mL ME α-mylase activity: IC_50_ = 15.7 mg/mL	(37)
*Trametes pubescens and Ganoderma adspersum*	Methanol extract (ME)	Not specified	*In vitro*	Both ME showed higher inhibitory activity on a-glucosidase enzyme than acarbose	IC_50_ (mg/mL) values for α-amylase inhibition: *T. pubescens* 0.12, *G. adspersum* 0.20, Acarbose 0.37	(37)
*Daedalea quercina*, *Hydnum repandum* and *Schizophyllum commune*	Metanol (ME) and hexan (HE) extracts of fruiting bodies	Phenolic compounds	*In vitro*	α-amylase inhibitory activity: *H. repandum* HE, *D. quercina* HE, *S. commune* HE, and *D. quercina* ME. α-glucosidase inhibitory activity: *D. quercina* ME	IC_50_ (mg/mL) values for α-amylase inhibition: *H. repandum* HE-0.25, *D. quercina* HE- 0.33, *S. commune* HE −0.42, and *D. quercina* ME-0.46. IC_50_ (mg/mL) value for α-glucosidase inhibition: *D. quercina* ME- 0.13.	(233)

## 7 Extraction of bioactive compounds

The utilization of the biological potential of plants and mushrooms is closely related to the method of processing and the preparation techniques used for their extracts, tinctures, essential oils, etc. Today, a wide range of extraction methods are available, many of which are capable of achieving high yields and preserving the bioactivity of target compounds. Through careful optimization of these processes, the presence of undesirable or interfering substances can be minimized or entirely avoided. Conventional extraction methods, such as maceration, Soxhlet extraction and percolation, are based on the principles of mass transfer. These processes typically begin with desorption of the analyte from the plant or fungal matrix, followed by diffusion of the desired compound through the organic layer of the matrix and its movement to the surface of the matrix-fluid interface. The analyte then disperses into the extraction phase, where it diffuses through the extraction medium and enters the region where convection occurs. A critical determinant of extraction efficiency is the understanding of the analyte’s physicochemical properties and its interactions with the matrix.

Traditional methods often rely on elevated temperatures to facilitate the release of bound analytes. In contrast, modern extraction techniques enhance process efficiency by introducing additional effects that impact the matrix or modulate the properties of the solvent. The most commonly used modern extraction techniques for obtaining extracts include ultrasonic (UAE), microwave (MAE), pressurized fluid (PLE) and supercritical fluid extraction (SCFE). These techniques operate with specific effects that contribute to their efficiency. Compared to traditional techniques, modern extraction methods are generally more time- and energy-efficient, and they typically employ environmentally friendly (“green”) solvents. As a result, the extracts obtained are well-suited for applications in the food and pharmaceutical industries. Recent advancements in novel extraction technologies, such as ultrasound-assisted extraction (180, 181), microwave-assisted extraction (182, 183), and extraction using natural deep eutectic solvents (26, 184, 185), have significantly improved the efficiency and yield of polyphenols from plant sources. These methods not only enhance extraction rates but also help preserve the integrity of the compounds, minimizing degradation and maximizing bioavailability. Consequently, they offer promising opportunities for the sustainable and effective recovery of polyphenols, facilitating their application in nutraceuticals, pharmaceuticals, and functional foods. Advances in extraction techniques, such as supercritical CO₂ extraction and microwave-assisted methods (75, 186, 187), have improved the recovery of these bioactive compounds from both plant and fungal sources, underscoring their potential applications in the nutraceutical, cosmetic, and pharmaceutical industries. UAE is a widespread modern technique commonly used for the preparation of extracts with strong antidiabetic effect. The mechanism of this technique is based on the generation of cavitation, which disrupts cell walls and facilitates the distribution of analytes from the pores of the matrix (180, 188). MAE uses microwave energy to generate heat through ionic conduction, disrupting cell walls and enhancing the release of bioactive compounds. This process increases collision frequency and internal pressure, leading to cell rupture and improved solvent diffusion. On the other hand, extractions under pressure affect the properties of the solvents themselves by changing their polarity, viscosity and diffusivity, thus improving the efficiency of the process. However, prolonged extraction time can favor greater absorption of microwave energy and thermal accumulation in the medium, increasing the risk of degradation of thermolabile compounds. Therefore, careful optimization of parameters such as microwave power, extraction time and solvent composition is essential to maximize the yield and stability of the target compounds (189). The SCFE technique is both traditional and innovative, and is favored for various applications due to its reliability, efficiency, and adaptability in industrial settings. The mild extraction conditions used in SCFE help to preserve the bioactivity and structural integrity of the extracted compounds.

The chemical composition of mushroom extracts – in particular the type and concentration of bioactive compounds – and their corresponding biological activities are influenced by the choice of solvent and extraction method. Studies have shown that alcoholic extracts (methanolic and ethanolic) of mushrooms are particularly rich in low molecular weight components and phenolic compounds (190). The preparation of mushroom extracts or bioactive compounds from mushroom fruiting bodies or mycelia involves several steps, namely pretreatment, isolation and purification (36). Mushroom extracts are often obtained in unpurified form and typically consist of a mixture of various bioactive compounds with high potential for the treatment of diabetes. The production of such extracts is generally more cost-effective than isolating a single pure compound, highlighting the advantage of using whole mushroom extract. Extraction is a crucial step to achieve the desired yield and bioactivity of isolated compounds. Water is widely used as a universal solvent due to its ability to dissolve a wide range of substances, along with its low cost and ease to handling. The hot water extraction (HWE) method is most commonly used for extracting water-soluble polysaccharides, as well as complexes of heteropolysaccharides and proteins from the outer glycoprotein layer of the cell wall. Prior to HWE, organic solvents and alcohol pretreatments are required to remove fats or low molecular weight impurities. The HWE process typically involves high temperatures (50–100°C) and extended extraction times (1.5–5 h). Increasing the temperature enhances extraction efficiency to a certain extent when the yield or purity starts to decrease beyond a certain threshold. Temperatures above 100°C may lead to *β*-glucan degradation, while higher temperatures (above 150°C) can disrupt the triple-helix structure and thus cause a decrease in bioactivity. A longer extraction time can increase yields, but also raise concerns about the economic sustainability and energy efficiency. Following HWE, alkaline or acidic extraction steps are usually performed to maximize the yield by hydrolyzing *β*-glucan and protein bonds in the middle layer of the cell wall, thereby isolating alkali- and/or acid-soluble fractions. Further processing steps to obtain pure compounds from crude polysaccharide extracts typically include ethanol precipitation, deproteinization, decolorization, dialysis and fractionation (191). Ultrasound-assisted extraction (UAE) has demonstrated superior efficiency for polysaccharide extraction from *Inonotus obliquus*, yielding a higher quantity (3.25%) and purity (73.16%) compared to traditional HWE (192). MAE and PLE have been evaluated for the extraction of polysaccharides, particularly *β*-glucans, from the fruiting bodies of *P. ostreatus* and *G. lucidum*. These studies confirm that extraction temperature is a key factor influencing polysaccharide yield when using MAE and PLE, with higher temperatures generally improving outcomes. However, variations in β-glucan content between extracts suggest that optimal extraction conditions must be tailored to each fungal species (193). SCFE is particularly effective for the extraction of lipophilic substances such as fatty acids and ergosterol from various mushroom species, often yielding higher purity and larger quantities compared to conventional solvent-based methods (194–196). The addition of co-solvents such as ethanol, methanol or water can enhance the extraction of polar compounds (e.g., phenols, flavonoids or polysaccharides), although it may reduce selectivity (197). Therefore, the choice and concentration of co-solvent must be carefully optimized to achieve an appropriate balance between extracting target compounds and minimizing undesired ones.

In a study conducted by Cvetanović Kljakić et al. (198), the influence of 7 different extraction techniques (accelerated solvent extraction; HAE: homogenizer-assisted extraction; MAE; maceration; SFE: supercritical CO_2_ extraction; SOX: soxhlet extraction; UAE) on the ability of the obtained extracts of *Glaucosciadium cordifolium* to inhibit amylase and glucosidase was examined. The results revealed significant differences among the extracts, and their ability to inhibit amylase ranged from 0.34 to 0.71 mmol ACAE/g. For glucosidase inhibition, the values obtained were in the range of 1.64–1.71 mmol ACAE/g, which is in favor of the SFE technique. Duran et al. (199) investigated the influence of different solvents and extraction techniques on the antidiabetic activity of two *Corydalis* species from Turkey. Ethanol extracts of *Corydalis erdelii* and *Corydalis solida* were obtained using the HAE technique, while the infusions were prepared with water. For both species, the HAE technique in combination with ethanol resulted in extracts with significantly higher antidiabetic activity. Specifically, for *C. erdelii*, the *α*-amylase inhibitory activity was 0.39 mmol ACAE/g for the ethanolic HAE extract versus 0.05 mmol ACAE/g for the aqueous infusion, while α-glucosidase inhibition was 0.99 mmol ACAE/g for the HAE extract, and absent in the infusion. A similar pattern was observed for *C. solida*, with amylase inhibition of 0.43 mmol ACAE/g for the HAE extract and 0.05 mmol ACAE/g for the infusion, while glucosidase inhibition was only observed in the HAE extract (0.87 mmol ACAE/g). Essential oils and extracts rich in non-polar components show considerable differences in antidiabetic activity depending on the extraction procedure. In their study on the ability of chamomile to inhibit amylase, Zengin et al. (72) demonstrated that extracts obtained via traditional SOX with hexane had a significantly lower affinity to inhibit this enzyme (0.66 mmol ACAE/g) compared to those obtained via microwave-assisted hydrodistillation (1.56 mmol ACAE/g). Furthermore, extraction conditions themselves influence both the composition and bioactivity of the extracts. For instance, SFE performed under different pressures (100–400 bar) yielded chamomile extracts with varying glucosidase inhibition values, ranging from 13.96 to 14.16 mmol ACAE/g (72).

## 8 Conclusion

T2DM now affects more than 11% of adults worldwide, causing over three million deaths annually, leading to significant healthcare costs. Its growing prevalence is approaching pandemic levels. In most cases, T2DM is linked to modern lifestyle factors including unhealthy diets, obesity and physical inactivity. Along with medical treatment, effective diabetes management requires ongoing support and education to achieve better health outcomes and quality of life.

Although synthetic drugs for diabetes are commercially available, their use is often limited by side effects and potential toxic effects on healthy cells. Therefore, medicinal plants and mushrooms have gained attention as a valuable source of bioactive primary and secondary metabolites, offering promising alternatives for the development of new therapeutics. Numerous studies have investigated their potential to reduce hyperglycemia through various mechanisms, including inhibition of *α*-glucosidase and α-amylase enzymes, enhancement of pancreatic *β*-cell function, modulation of antioxidant defense systems, regulation of carbohydrate metabolism and stimulation of insulin secretion. However, the broader application of natural products in therapy requires not only the identification of the active compounds and clarification of their mechanisms of action, but also the development of standardized production protocols and quality control measures quality.

The WHO Global Traditional Medicine Centre was established to generate evidence and data that support the development of standards and regulatory frameworks, ensuring the safe, cost-effective and equitable use of traditional medicine. Its mission is to optimize the role of traditional medicine in global health and sustainable development, with a focus on evidence, innovation and sustainability (7). Additionally, the WHO launched the International Regulatory Cooperation for Herbal Medicines to improve the global regulation of herbal products (200). WHO also recognized traditional and complementary medicine through achieving Sustainable Development Goal 3: “Ensure healthy lives and promote well-being for all at all ages” (4). It is important to note that the presence of multiple components in natural extracts can play a crucial role in their therapeutic potential. The components may act synergistically to enhance efficacy or antagonistically, potentially reducing the desired effect.

The choice of extraction method and extraction parameters (e.g., type of solvent, temperature, time) has a significant impact on the chemical composition and concentration of the extracted bioactive components, which in turn directly affects their biological and therapeutic activity. Therefore, improving the selectivity of the extraction by selecting the most suitable solvents and techniques is essential for isolating the desired component from the mixture while minimizing interfering components. It is the researcher’s responsibility to select appropriate solvents and apply optimal extraction techniques to isolate the target bioactive compounds while reducing the presence of unwanted or interfering substances. In addition to enhancing therapeutic quality, interactions with pharmaceutical extracts must be further investigated and clarified to ensure consistency and safety. Moreover, the successful integration of natural extracts into therapeutic practice requires an approach that addresses several critical aspects. One of the primary priorities is determining the effective dosage to maximize therapeutic benefit while minimizing the risk of adverse effects. Equally important is the evaluation of pharmacokinetic parameters to understand the bioavailability and therapeutic efficacy of natural products through their absorption, distribution, metabolism and excretion. The development of regulations and standardization protocols is essential to ensure the quality, consistency and safety of the natural extracts or compounds used in a therapy. Currently, most of the *in vivo* evidence supporting the therapeutic properties of natural antidiabetic extracts and compounds, particularly those derived from mushrooms, is based on animal studies. Although animal studies provide valuable preliminary evidence, they are not sufficient to establish reliable efficacy and safety for use in human therapy. Therefore, well-designed clinical studies, coupled with a deeper understanding of the mechanisms of action and therapeutic efficacy, are essential to support the use of natural extracts in diabetes treatment. Despite the many challenges and existing gaps, the combined knowledge of traditional medicine and modern science holds great potential to contribute to modern health and food systems. This integration can facilitate the translation of traditional practices into practical applications and commercial products, laying the foundation for the development of novel foods, medicines and nutraceuticals.

## References

[1] Fabricant DS Farnsworth NR. The value of plants used in traditional medicine for drug discovery. Environ Health Perspect. (2001) 109:69–75. doi: 10.1289/ehp.01109s169, PMID: 11250806 PMC1240543

[2] Lee KH Morris-Natschke S Yang X Huang R Zhou T Wu SF. Recent progress of research on medicinal mushrooms, foods, and other herbal products used in traditional Chinese medicine. J Tradit Complement Med. (2012) 2:1–12. doi: 10.1016/s2225-4110(16)30081-5, PMID: 24716120 PMC3942920

[3] Yuan H Ma Q Ye L Piao G. The traditional medicine and modern medicine from natural products. Molecules. (2016) 21:559. doi: 10.3390/molecules21050559, PMID: 27136524 PMC6273146

[4] WHO. (2019). WHO global report on traditional and complementary medicine 2019. World Health Organisation 1–228. Available online at: https://apps.who.int/iris/bitstream/handle/10665/312342/9789241515436-eng.pdf?ua=1 (Accessed June 26, 2024).

[5] Rosén J Gottfries J Muresan S Backlund A Oprea TI. Novel chemical space exploration via natural products. J Med Chem. (2009) 52:1953–62. doi: 10.1021/jm801514w, PMID: 19265440 PMC2696019

[6] Ngo LT Okogun JI Folk WR. 21st century natural product research and drug development and traditional medicines. Nat Prod Rep. (2013) 30:584–92. doi: 10.1039/c3np20120a, PMID: 23450245 PMC3652390

[7] World Health Organization (WHO). About us: WHO global traditional medicine centre. Available online at: https://www.who.int/initiatives/who-global-traditional-medicine-centre/about-us%0A%0A (Accessed June 26, 2024).

[8] De Silva DD Rapior S Hyde KD Bahkali AH. Medicinal mushrooms in prevention and control of diabetes mellitus. Fungal Divers. (2012) 56:1–29. doi: 10.1007/s13225-012-0187-4

[9] Weber S. (2021). IDF Diabetes Atlas 10th Edition. Available online at: https://diabetesatlas.org/ (Accessed June 19, 2024).

[10] Tripathy B Sahoo N Sahoo SK. Trends in diabetes care with special emphasis to medicinal plants: advancement and treatment. Biocatal Agric Biotechnol. (2021) 33:102014–1. doi: 10.1016/j.bcab.2021.102014, PMID: 35342487 PMC8941016

[11] American Diabetes Association. Diagnosis and classification of diabetes mellitus. Diabetes Care. (2014) 37:S81–90. doi: 10.2337/dc14-S081, PMID: 24357215

[12] International Diabetes Federation. IDF Diabetes Atlas 2025. Available online at: https://diabetesatlas.org/resources/idf-diabetes-atlas-2025/ (Accessed June 19, 2024).

[13] Kahn SE Hull RL Utzschneider KM. Mechanisms linking obesity to insulin resistance and type 2 diabetes. Nature. (2006) 444:840–6. doi: 10.1038/nature05482, PMID: 17167471

[14] Xiaofei L Donghui L Jingjing G Jin C Xiaofei X. Mushroom polysaccharides with potential in anti-diabetes: biological mechanisms, extraction, and future perspectives: a review Xiaofei. Front Nutr. (2022) 9:1087826. doi: 10.3389/fnut.2022.1087826, PMID: 36590224 PMC9794872

[15] Jugran AK Rawat S Devkota HP Bhatt ID Rawal RS. Diabetes and plant-derived natural products: from ethnopharmacological approaches to their potential for modern drug discovery and development. Phytother Res. (2021) 35:223–45. doi: 10.1002/ptr.6821, PMID: 32909364

[16] Database p. national library of medicine. Available online at: https://pubmed.ncbi.nlm.nih.gov/ (Accessed June 19, 2024).

[17] Choi CI. Sodium-glucose cotransporter 2 (SGLT2) inhibitors from natural products: discovery of next-generation antihyperglycemic agents. Molecules. (2016) 21:1–12. doi: 10.3390/molecules21091136 PMC6273509 27618891

[18] Kour H Kour D Kour S Singh S Jawad Hashmi SA Yadav AN. Bioactive compounds from mushrooms: emerging bioresources of food and nutraceuticals. Food Biosci. (2022) 50:102124–18. doi: 10.1016/j.fbio.2022.102124, PMID: 40399227

[19] Mushroom Wisdom I. SX-FRACTION®, 90 Tablets. Available online at: https://mushroomwisdom.com/product/sx-fraction-90-tablets/ (Accessed June 19, 2024).

[20] Butler MS Robertson AAB Cooper MA. Natural product and natural product derived drugs in clinical trials. Nat Prod Rep. (2014) 31:1612–61. doi: 10.1039/c4np00064a, PMID: 25204227

[21] Astra Zeneca. FORXIGA™ (dapagliflozin) now approved in European Union for treatment of type 2 diabetes. Available online at: https://www.astrazeneca.com/media-centre/press-releases/2012/FORXIGA-dapagliflozin-now-approved-in-European-Union-for-treatment-of-type-2-diabetes-14112012.html#! (Accessed June 17, 2024).

[22] AstraZeneca. Farxiga approved in the US for the treatment of paediatric type-2 diabetes. Available online at: https://www.astrazeneca.com/media-centre/press-releases/2024/farxiga-approved-in-the-us-for-the-treatment-of-paediatric-type-2-diabetes.html (Accessed June 17, 2024).

[23] Bailey CJ. Metformin: historical overview. Diabetologia. (2017) 60:1566–76. doi: 10.1007/s00125-017-4318-z, PMID: 28776081

[24] Yang JP Hsu T Lin F Hsu W Chen Y. Potential antidiabetic activity of extracellular polysaccharides in submerged fermentation culture of Coriolus versicolor LH1. Carbohydr Polym. (2012) 90:174–80. doi: 10.1016/j.carbpol.2012.05.011, PMID: 24751027

[25] Šovljanski O Cvetanović Kljakić A Tomić A. Antibacterial and antifungal potential of plant secondary metabolites. In: Mérillon J-M Ramawat KG, editors. Plant specialized metabolites: Phytochemistry, ecology and biotechnology. Cham: Springer (2023). 1–43.

[26] Pavlić B Aćimović M Sknepnek A Miletić D Mrkonjić Ž Kljakić AC. Sustainable raw materials for efficient valorization and recovery of bioactive compounds. Ind Crop Prod. (2023) 193:116167. doi: 10.1016/J.INDCROP.2022.116167

[27] Ansari P Samia JF Khan JT Rafi MR Rahman MS Rahman AB. Protective effects of medicinal plant-based foods against diabetes: a review on pharmacology, phytochemistry, and molecular mechanisms. Nutrients. (2023) 15:1–41. doi: 10.3390/nu15143266 PMC10383178 37513684

[28] Wojdyło A Nowicka P Grimalt M Legua P Almansa MS Amorós A. Polyphenol compounds and biological activity of caper (*Capparis spinosa* l.) flowers buds. Plan Theory. (2019) 8:539. doi: 10.3390/plants8120539, PMID: 31775254 PMC6963175

[29] Rahman MS. Allicin and other functional active components in garlic: health benefits and bioavailability. Int J Food Prop. (2007) 10:245–68. doi: 10.1080/10942910601113327

[30] Pereira CG Barreira L Bijttebier S Pieters L Neves V Rodrigues MJ. Chemical profiling of infusions and decoctions of Helichrysum italicum subsp. picardii by UHPLC-PDA-MS and in vitro biological activities comparatively with green tea (*Camellia sinensis*) and rooibos tisane (*Aspalathus linearis*). J Pharm Biomed Anal. (2017) 145:593–603. doi: 10.1016/J.JPBA.2017.07.007, PMID: 28787672

[31] Sun C Zhao C Guven EC Paoli P Simal-Gandara J Ramkumar KM. Dietary polyphenols as antidiabetic agents: advances and opportunities. Food Front. (2020) 1:18–44. doi: 10.1002/fft2.15

[32] Miletić D Pantić M Sknepnek A Vasiljević I Lazović M Nikšić M. Influence of selenium yeast on the growth, selenium uptake and mineral composition of *Coriolus versicolor* mushroom. J Basic Microbiol. (2020) 60:331–40. doi: 10.1002/jobm.201900520, PMID: 32003038

[33] Miletić D Turło J Podsadni P Pantić M Nedović V Lević S. Selenium-enriched *Coriolus versicolor* mushroom biomass: potential novel food supplement with improved selenium bioavailability. J Sci Food Agric. (2019) 99:5122–30. doi: 10.1002/jsfa.9756, PMID: 30993725

[34] Rašeta M Popović M Čapo I Stilinović N Vukmirović S Milošević B. Antidiabetic effect of two different: Ganoderma species tested in alloxan diabetic rats. RSC Adv. (2020) 10:10382–93. doi: 10.1039/c9ra10158f, PMID: 35498606 PMC9050389

[35] Xiong M Huang Y Liu Y Huang M Song G Ming Q. Antidiabetic activity of ergosterol from *Pleurotus ostreatus* in KK-ay mice with spontaneous type 2 diabetes mellitus. Mol Nutr Food Res. (2018) 62:1700444. doi: 10.1002/mnfr.201700444 29080247

[36] Huang CH Lin WK Chang S Tsai GJ. Evaluation of the hypoglycaemic and antioxidant effects of submerged *Ganoderma lucidum* cultures in type 2 diabetic rats. Mycology. (2021) 12:82–93. doi: 10.1080/21501203.2020.1733119, PMID: 34026300 PMC8128183

[37] Deveci E Çayan F Tel-Çayan G Duru ME. Inhibitory activities of medicinal mushrooms on α-amylase and α-glucosidase-enzymes related to type 2 diabetes. S Afr J Bot. (2021) 137:19–23. doi: 10.1016/j.sajb.2020.09.039

[38] Wu Q Xiao C Yang X Zhang J. Hypoglycemic effects of components extracted from edible and medicinal fungi and their mechanisms of action. Acta Edulis Fungi. (2009) 16:80–6. doi: 10.16488/j.cnki.1005-9873.2009.03.020

[39] Ma B Feng T Zhang S Zhuang H Chen D Yao L. The inhibitory effects of Hericium erinaceus β-glucan on in vitro starch digestion. Front Nutr. (2021) 7:621131. doi: 10.3389/fnut.2020.621131, PMID: 33553235 PMC7859327

[40] Sknepnek A Miletić D. “Application of mushrooms in beverages,” In: Deshmukl SK Sridhar KR Badalyan SM, editors. Fungal biotechnology: Prospects and avenue. London, England: CRC Press (2022) 280–309.

[41] Zhang H Qui M Chen Y Chen J Sun Y Wang C. “Plant terpenes. Phytochemistry and Pharmacognosy,” In: Malenčić P, editor. Encyclopedia of life support systems. Oxford, United Kingdom: EOLSS Publishers. (2011).

[42] Grbović S. Hemijska karakterizacija i biološke aktivnosti vrsta *Eucalyptus camaldulensis* Dehnh. i Eukalyptus gunni Hook. iz Crne Gore. [Doctoral dissertation]. Novi Sad: Univerzitet u Novom Sadu, Prirodno-matematički fakultet. (2010) 182.

[43] Macheix J-J. Fruit phenolics. 1st Editio ed. Boca Raton, Florida, USA: CRC Press (1990). 390 p.

[44] Strack D. Phenolic metabolism. In: Dey PM Harbrorne PM, editors. Plant biochemistry. New York, USA: Academic Press (1997)

[45] Dixon RA Paiva NL. Stress-induced phenylpropanoid metabolism. Plant Cell. (1995) 7:1085–97. doi: 10.2307/3870059, PMID: 12242399 PMC160915

[46] Valanciene E Jonuskiene I Syrpas M Augustiniene E Matulis P Simonavicius A. Advances and prospects of phenolic acids production, biorefinery and analysis. Biomol Ther. (2020) 10:1–41. doi: 10.3390/biom10060874 PMC7356249 32517243

[47] Raina J Firdous A Singh G Kumar R Kaur C. Role of polyphenols in the management of diabetic complications. Phytomedicine. (2024) 122:155155. doi: 10.1016/j.phymed.2023.155155, PMID: 37922790

[48] Pan D Zhang D Wu J Chen C Xu Z Yang H. Antidiabetic, Antihyperlipidemic and antioxidant activities of a novel proteoglycan from Ganoderma Lucidum fruiting bodies on db/db mice and the possible mechanism. PLoS One. (2013) 8:e68332. doi: 10.1371/journal.pone.0068332, PMID: 23874589 PMC3708940

[49] Liang B Guo Z Xie F Zhao A. Antihyperglycemic and antihyperlipidemic activities of aqueous extract of Hericium erinaceus in experimental diabetic rats. BMC Complement Altern Med. (2013) 13:253. doi: 10.1186/1472-6882-13-253, PMID: 24090482 PMC3852124

[50] Sknepnek A Pantić M Matijašević D Miletić D Lević S Nedović V. Novel kombucha beverage from lingzhi or reishi medicinal mushroom, Ganoderma lucidum, with antibacterial and antioxidant effects. Int J Med Mushrooms. (2018) 20:243–58. doi: 10.1615/intjmedmushrooms.2018025833, PMID: 29717669

[51] Miletić D Turło J Podsadni P Sknepnek A Szczepańska A Lević S. Turkey tail medicinal mushroom, Trametes versicolor (Agaricomycetes), crude exopolysaccharides with antioxidative activity. Int J Med Mushrooms. (2020) 22:885–95. doi: 10.1615/intjmedmushrooms.2020035877, PMID: 33389854

[52] Miletić D Turło J Podsadni P Sknepnek A Szczepańska A Klimaszewska M. Production of bioactive selenium enriched crude exopolysaccharides via selenourea and sodium selenite bioconversion using *Trametes versicolor*. Food Biosci. (2016) 16:66–71. doi: 10.1016/j.fbio.2021.101046

[53] Stojanova M Pantić M Karadelev M Čuleva B Nikšić M. Antioxidant potential of extracts of three mushroom species collected from the republic of North Macedonia. J Food Process Preserv. (2021) 45:e15155. doi: 10.1111/jfpp.15155

[54] de Paulo Farias D Araújo FF Neri-Numa IA Pastore GM. Antidiabetic potential of dietary polyphenols: a mechanistic review. Food Res Int. (2021) 145:110383. doi: 10.1016/j.foodres.2021.110383, PMID: 34112386

[55] Junejo JA Zaman K Rudrapal M Celik I Attah EI. Antidiabetic bioactive compounds from *Tetrastigma angustifolia* (Roxb.) deb and *Oxalis debilis* Kunth.: validation of ethnomedicinal claim by in vitro and in silico studies. S Afr J Bot. (2021) 143:164–75. doi: 10.1016/j.sajb.2021.07.023

[56] Pandey KB Rizvi SI. Plant polyphenols as dietary antioxidants in human health and disease. Oxidative Med Cell Longev. (2009) 2:270–8. doi: 10.4161/oxim.2.5.9498, PMID: 20716914 PMC2835915

[57] Cvetanović A Švarc-Gajić J Gašić U Tešić Ž Zengin G Zeković Z. Isolation of apigenin from subcritical water extracts: optimization of the process. J Supercrit Fluids. (2017) 120:32–42. doi: 10.1016/j.supflu.2016.10.012

[58] Im KH Nguyen TK Choi J Lee TS. In vitro antioxidant, anti-diabetes, anti-dementia, and inflammation inhibitory effect of Trametes pubescens fruiting body extracts. Molecules. (2016) 21:639. doi: 10.3390/molecules21050639, PMID: 27196881 PMC6273937

[59] Wu T Xu B. Antidiabetic and antioxidant activities of eight medicinal mushroom species from China. (2015). 129–140. doi: 10.1615/intjmedmushrooms.v17.i2.40 25746618

[60] Gacche RN Dhole NA. Profile of aldose reductase inhibition, anti-cataract and free radical scavenging activity of selected medicinal plants: an attempt to standardize the botanicals for amelioration of diabetes complications. Food Chem Toxicol. (2011) 49:1806–13. doi: 10.1016/J.FCT.2011.04.032, PMID: 21570444

[61] Fatmawati S Kurashiki K Takeno S Kim Y Shimizu K Sato M. The inhibitory effect on aldose reductase by an extract of *Ganoderma lucidum*. Phytother Res. (2009) 23:28–32. doi: 10.1002/ptr.2425, PMID: 19107825

[62] Abdelshafy AM Belwal T Liang Z Wang L Li D Luo Z. A comprehensive review on phenolic compounds from edible mushrooms: occurrence, biological activity, application and future prospective. Crit Rev Food Sci Nutr. (2022) 62:6204–24. doi: 10.1080/10408398.2021.1898335, PMID: 33729055

[63] Kalita D Holm DG LaBarbera DV Petrash JM Jayanty SS. Inhibition of α-glucosidase, α-amylase, and aldose reductase by potato polyphenolic compounds. PLoS One. (2018) 13:e0191025. doi: 10.1371/journal.pone.0191025, PMID: 29370193 PMC5784920

[64] Kang GG Francis N Hill R Waters D Blanchard C Santhakumar AB. Dietary polyphenols and gene expression in molecular pathways associated with type 2 diabetes mellitus: a review. Int J Mol Sci. (2019) 21:140. doi: 10.3390/ijms21010140, PMID: 31878222 PMC6981492

[65] Câmara JS Perestrelo R Ferreira R Berenguer CV Pereira JAM Castilho PC. Plant-derived Terpenoids: a plethora of bioactive compounds with several health functions and industrial applications—a comprehensive overview. Molecules. (2024) 29:3861. doi: 10.3390/molecules29163861, PMID: 39202940 PMC11357518

[66] Panigrahy SK Bhatt R Kumar A. Targeting type II diabetes with plant terpenes: the new and promising antidiabetic therapeutics. Biologia. (2021) 76:241–54. doi: 10.2478/s11756-020-00575-y

[67] Jeppesen PB Gregersen S Poulsen CR Hermansen K. Stevioside acts directly on pancreatic β cells to secrete insulin: actions independent of cyclic adenosine monophosphate and adenosine triphosphate—sensitivie K+-channel activity. Metabolism. (2000) 49:208–14. doi: 10.1016/S0026-0495(00)91325-8, PMID: 10690946

[68] Lailerd N Saengsirisuwan V Sloniger JA Toskulkao C Henriksen EJ. Effects of stevioside on glucose transport activity in insulin-sensitive and insulin-resistant rat skeletal muscle. Metabolism. (2004) 53:101–7. doi: 10.1016/j.metabol.2003.07.014, PMID: 14681850

[69] Atri NS Sharma SK Joshi R Gulati A Gulati A. Nutritional and neutraceutical composition of five wild culinary-medicinal species of genus Pleurotus (higher Basidiomycetes) from Northwest India. Int J Med Mushrooms. (2013) 15:49–56. doi: 10.1615/intjmedmushr.v15.i1.60, PMID: 23510284

[70] Mbaze LM Poumale HMP Wansi JD Lado JA Khan SN Iqbal MC. Α-Glucosidase inhibitory pentacyclic triterpenes from the stem bark of Fagara tessmannii (Rutaceae). Phytochemistry. (2007) 68:591–5. doi: 10.1016/j.phytochem.2006.12.015, PMID: 17270224

[71] Hou W Li Y Zhang Q Wei X Peng A Chen L. Triterpene acids isolated from *Lagerstroemia speciosa* leaves as α-glucosidase inhibitors. Phytother Res. (2009) 23:614–8. doi: 10.1002/ptr.2661, PMID: 19107840

[72] Zengin G Mollica A Arsenijević J Pavlić B Zeković Z Sinan KI. A comparative study of chamomile essential oils and lipophilic extracts obtained by conventional and greener extraction techniques: Chemometric approach to chemical composition and biological activity. Separations. (2023) 10:18. doi: 10.3390/separations10010018, PMID: 40278680

[73] Zhao XR Huo XK Dong PP Wang C Huang SS Zhang BJ. Inhibitory effects of highly oxygenated lanostane derivatives from the fungus *Ganoderma lucidum* on P-glycoprotein and α-glucosidase. J Nat Prod. (2015) 78:1868–76. doi: 10.1021/acs.jnatprod.5b00132, PMID: 26222905

[74] Chen XQ Zhao J Chen LX Wang SF Wang Y Li SP. Lanostane triterpenes from the mushroom Ganoderma resinaceum and their inhibitory activities against α-glucosidase. Phytochemistry. (2018) 149:103–15. doi: 10.1016/j.phytochem.2018.01.007, PMID: 29490285

[75] Dasgupta A Acharya K. Mushrooms: an emerging resource for therapeutic terpenoids. 3 Biotech. (2019) 9:1–14. doi: 10.1007/s13205-019-1906-2 PMC6760460 31588393

[76] Fatmawati S Kondo R Shimizu K. Structure-activity relationships of lanostane-type triterpenoids from *Ganoderma lingzhi* as α-glucosidase inhibitors. Bioorg Med Chem Lett. (2013) 23:5900–3. doi: 10.1016/j.bmcl.2013.08.084 24070782

[77] Sato M Tai T Nunoura Y Yajima Y Kawashima S Tanaka K. Dehydrotrametenolic acid induces preadipocyte differentiation and sensitizes animal models of noninsulin-dependent diabetes mellitus to insulin. Biol Pharm Bull. (2002) 25:81–6. doi: 10.1248/bpb.25.81, PMID: 11824563

[78] Yoshikawa M Murakami T Shimada H Matsuda H Yamahara J Tanabe G. Salacinol, potent antidiabetic principle with unique thiosugar sulfonium sulfate structure from the ayurvedic traditional medicine *Salacia reticulata* in Sri Lanka and India. Tetrahedron Lett. (1997) 38:8367–70. doi: 10.1016/S0040-4039(97)10270-2

[79] Yoshikawa M Murakami T Yashiro K Matsuda H. Kotalanol, a potent a-glucosidase inhibitor with Thiosugar Sulfonium sulfate structure, from antidiabetic Ayurvedic medicine Salacia reticulata. Chem Pharm Bull. (1998) 46:1339–40. doi: 10.1248/cpb.46.1339, PMID: 9734318

[80] Yoshikawa M Morikawa T Matsuda H Tanabe G Muraoka O. Absolute stereostructure of potent α-glucosidase inhibitor, salacinol, with unique thiosugar sulfonium sulfate inner salt structure from *Salacia reticulata*. Bioorg Med Chem. (2002) 10:1547–54. doi: 10.1016/S0968-0896(01)00422-9, PMID: 11886816

[81] Yoshikawa M Ninomya K Shimoda H Nishida N Matsuda H. Hepatoprotective and antioxidative properties of *Salacia reticulata*: preventive effects of phenolic constituents on CCl4-induced liver injury in mice. Biol Pharm Bull. (2002) 25:72–6. doi: 10.1248/bpb.25.72 11824561

[82] Yoshikawa M Shimoda H Nishida N Takada M Matsuda H. *Salacia reticulata* and its polyphenolic constituents with lipase inhibitory and lipolytic activities have mild antiobesity effects in rats. J Nutr. (2002) 132:1819–24. doi: 10.1093/jn/132.7.1819, PMID: 12097653

[83] Matsuda H Murakami T Yashiro K Yamahara J Yoshikawa M. Antidiabetic principles of natural medicines. IV. Aldose reductase and α-glucosidase inhibitors from the roots of *Salacia oblonga* WALL. (Celastraceae): structure of a new friedelane-type triterpene, kotalagenin 16-acetate. Chem Pharm Bull. (1999) 47:1725–9. doi: 10.1248/cpb.47.1725 10748716

[84] Yoshikawa M Pongpiriyadacha Y Kishi A Kageura T Wang T Morikawa T. Biological activities of *Salacia chinensis* originating in Thailand: the quality evaluation guided by α-glucosidase inhibitory activity. Yakugaku Zasshi. (2003) 123:871–80. doi: 10.1248/yakushi.123.871, PMID: 14577333

[85] Morikawa T Kishi A Pongpiriyadacha Y Matsuda H Yoshikawa M. Structures of new friedelane-type triterpenes and eudesmane-type sesquiterpene and aldose reductase inhibitors from *Salacia chinensis*. J Nat Prod. (2003) 66:1191–6. doi: 10.1021/np0301543, PMID: 14510595

[86] Praveen Kumar M Poornima ME Al-Ghanim K Al-Misned F Ahmed Z Mahboob S. Effects of D-limonene on aldose reductase and protein glycation in diabetic rats. J King Saud Univ Sci. (2020) 32:1953–8. doi: 10.1016/j.jksus.2020.01.043

[87] Wang HY Kan WC Cheng TJ Yu SH Chang LH Chuu JJ. Differential anti-diabetic effects and mechanism of action of charantin-rich extract of Taiwanese *Momordica charantia* between type 1 and type 2 diabetic mice. Food Chem Toxicol. (2014) 69:347–56. doi: 10.1016/j.fct.2014.04.008, PMID: 24751968

[88] Mishra A Gautam S Pal S Mishra A Rawat AK Maurya R. Effect of *Momordica charantia* fruits on streptozotocin-induced diabetes mellitus and its associated complications. Int J Pharm Pharm Sci. (2015) 7:356–63.

[89] Fallah Huseini H Hasani-Rnjbar S Nayebi N Heshmat R Sigaroodi FK Ahvazi M. *Capparis spinosa* L. (caper) fruit extract in treatment of type 2 diabetic patients: a randomized double-blind placebo-controlled clinical trial. Complement Ther Med. (2013) 21:447–52. doi: 10.1016/j.ctim.2013.07.003, PMID: 24050578

[90] Azadmehr A Ziaee A Ghanei L Huseini HF Hajiaghaee R Tavakoli-Far B. A randomized clinical trial study: anti-oxidant, anti-hyperglycemic and anti-hyperlipidemic effects of olibanum gum in type 2 diabetic patients. Iran J Pharm Res. (2014) 13:1003–10. 25276202 PMC4177622

[91] Bustanji Y Al-Masri IM Mohammad M Hudaib M Tawaha K Tarazi H. Pancreatic lipase inhibition activity of trilactone terpenes of *Ginkgo biloba*. J Enzyme Inhib Med Chem. (2011) 26:453–9. doi: 10.3109/14756366.2010.525509, PMID: 21028941

[92] Kang MS Hirai S Goto T Kuroyanagi K Kim YI Ohyama K. Dehdroabietic acid,a diterpene improves diabetes and hyperlipdemia in obese diabitic KK-ay mice. Biofactors. (2009) 35:442–8. doi: 10.1002/biof.58 19753653

[93] Rasouli H Yarani R Pociot F Popović-Djordjević J. Anti-diabetic potential of plant alkaloids: revisiting current findings and future perspectives. Pharmacol Res. (2020) 155:104723. doi: 10.1016/j.phrs.2020.104723, PMID: 32105756

[94] Kutchan TM. Alkaloid biosynthesis – the basis for metabolic engineering of medicinal plants. Plant Cell. (1995) 7:1059–70. doi: 10.2307/3870057, PMID: 12242397 PMC160910

[95] Boucher R Germain H Desgagné-penix I. Exploring the lesser-known bioactive natural products of plant species of the genus *Cannabis* L.: alkaloids, phenolic compounds, and their therapeutic potential. Plan Theory. (2025) 14:1372. doi: 10.3390/plants14091372, PMID: 40364401 PMC12073235

[96] Zorrilla JG Evidente A. Structures and biological activities of alkaloids produced by mushrooms, a fungal subgroup. Biomol Ther. (2022) 12:1–25. doi: 10.3390/biom12081025 PMC9332295 35892335

[97] van der Laar FA. Alpha-glucosidase inhibitors in the early treatment of type 2 diabetes. Vasc Health Risk Manag. (2008) 4:1189–95. doi: 10.2147/vhrm.s3119, PMID: 19337532 PMC2663450

[98] Rasouli H Hosseini-Ghazvini SMB Adibi H Khodarahmi R. Differential α-amylase/α-glucosidase inhibitory activities of plant-derived phenolic compounds: a virtual screening perspective for the treatment of obesity and diabetes. Food Funct. (2017) 8:1942–54. doi: 10.1039/c7fo00220c, PMID: 28470323

[99] Gopinath G Sankeshi V perugu S Alaparthi MD Bandaru S Pasala VK. Design and synthesis of chiral 2H-chromene-N-imidazolo-amino acid conjugates as aldose reductase inhibitors. Eur J Med Chem. (2016) 124:750–62. doi: 10.1016/j.ejmech.2016.08.070, PMID: 27639366

[100] Srivastava SK Ramana KV Bhatnagar A. Role of aldose reductase and oxidative damage in diabetes and the consequent potential for therapeutic options. Endocr Rev. (2005) 26:380–92. doi: 10.1210/er.2004-0028, PMID: 15814847

[101] Gupta S Singh N Jaggi AS. Alkaloids as aldose reductase inhibitors, with special reference to berberine. J Altern Complement Med. (2014) 20:195–205. doi: 10.1089/acm.2013.0088, PMID: 24236461

[102] Liang L Wu X Zhao T Zhao J Li F Zou Y. In vitro bioaccessibility and antioxidant activity of anthocyanins from mulberry (*Morus atropurpurea* Roxb.) following simulated gastro-intestinal digestion. Food Res Int. (2012) 46:76–82. doi: 10.1016/j.foodres.2011.11.024

[103] Tang B-Q Yang T Yang W Wang W Zhang X Ye W. Chemical constituents in leaves of *Morus atropurpurea* and their α-glucosidase activity. Chin Tradit Herb Drug. (2013) 44:3109–13. doi: 10.7501/j.issn.0253-2670.2013.22.003

[104] Asano N Oseki K Tomioka E Kizu H Matsui K. N-containing sugars from *Morus alba* and their glycosidase inhibitory activities. Carbohydr Res. (1994) 259:243–55. doi: 10.1016/0008-6215(94)84060-1, PMID: 8050098

[105] Asano N Tomioka E Kizu H Matsui K. Sugars with nitrogen in the ring isolated from the leaves of Morus bombycis. Carbohydr Res. (1994) 253:235–45. doi: 10.1016/0008-6215(94)80068-5, PMID: 8156550

[106] Tabussum A Riaz N Saleem M Ashraf M Ahmad M Alam U. Α-Glucosidase inhibitory constituents from *Chrozophora plicata*. Phytochem Lett. (2013) 6:614–9. doi: 10.1016/J.PHYTOL.2013.08.005

[107] Wang J Bi C Xi H Wei F. Effects of administering berberine alone or in combination on type 2 diabetes mellitus: a systematic review and meta-analysis. Front Pharmacol. (2024) 15:1455534. doi: 10.3389/fphar.2024.1455534, PMID: 39640489 PMC11617981

[108] Nguyen V Taine EG Meng D Cui T Tan W. Pharmacological activities, therapeutic effects, and mechanistic actions of Trigonelline. Int J Mol Sci. (2024) 25:3385. doi: 10.3390/ijms25063385, PMID: 38542359 PMC10970276

[109] Wan Y Yu Y Pan X Mo X Gong W Liu X. Inhibition on acid-sensing ion channels and analgesic activities of flavonoids isolated from dragon’s blood resin. Phytother Res. (2019) 33:718–27. doi: 10.1002/ptr.6262, PMID: 30618119

[110] Zhang X Jin Y Wu Y Zhang C Jin D Zheng Q. Anti-hyperglycemic and anti-hyperlipidemia effects of the alkaloid-rich extract from barks of Litsea glutinosa in Ob/Ob mice. Sci Rep. (2018) 8:12646. doi: 10.1038/s41598-018-30823-w, PMID: 30140027 PMC6107583

[111] Sharma B Salunke R Balomajumder C Daniel S Roy P. Anti-diabetic potential of alkaloid rich fraction from Capparis decidua on diabetic mice. J Ethnopharmacol. (2010) 127:457–62. doi: 10.1016/j.jep.2009.10.013, PMID: 19837152

[112] Kumar S Rashmi Kumar D. Evaluation of antidiabetic activity of *Euphorbia hirta* Linn. In streptozotocin induced diabetic mice. Indian J Nat Prod Resour. (2010) 1:200–3.

[113] Cvetanović Kljakić A Ocvirk M Rutnik K Košir IJ Pavlić B Mašković P. Exploring the composition and potential uses of four hops varieties through different extraction techniques. Food Chem. (2024) 447:138910. doi: 10.1016/j.foodchem.2024.138910, PMID: 38479143

[114] Jovanović JA Mihailović M Uskoković A Grdović N Dinić S Vidaković M. The effects of major mushroom bioactive compounds on mechanisms that control blood glucose level. J Fungi. (2021) 7:1–15. doi: 10.3390/jof7010058 PMC7830770 33467194

[115] Hu JL Nie SP Xie MY. Antidiabetic mechanism of dietary polysaccharides based on their gastrointestinal functions. J Agric Food Chem. (2018) 66:4781–6. doi: 10.1021/acs.jafc.7b05410, PMID: 29671596

[116] Mihailović M Jovanović JA Uskoković A Grdović N Dinić S Vidović S. Protective effects of the mushroom *Lactarius deterrimus* extract on systemic oxidative stress and pancreatic islets in streptozotocin-induced diabetic rats. J Diabetes Res. (2015) 2015:576726. doi: 10.1155/2015/576726 26221612 PMC4499631

[117] Ganesan K Xu B. Anti-diabetic effects and mechanisms of dietary polysaccharides. Molecules. (2019) 24:2556. doi: 10.3390/molecules24142556, PMID: 31337059 PMC6680889

[118] Nikam R Kiani H Mousavi ZE Mousavi M. Extraction, Detection, and Characterization of Various Chemical Components of *Trigonella foenum-graecum* L. (Fenugreek) Known as a Valuable Seed in Agriculture. In: Naeem M Aftab T Masroor MAK, editors. Fenugreek biology and application. Singapore: Springer (2021) 189–217.

[119] Qader M Xu J Yang Y Wu X Liu Y Cao S. Chemistry behind the immunomodulatory activity of *Astragalus membranaceus*. Chin Med Cult. (2021) 4:201–10. doi: 10.4103/CMAC.CMAC_40_21

[120] Alqudah SM Hailat M Zakaraya Z Abu Dayah AA Abu Assab M Alarman SM. Impact of *Opuntia ficus-indica* juice and empagliflozin on glycemic control in rats. Curr Issues Mol Biol. (2024) 46:12343–53. doi: 10.3390/cimb46110733, PMID: 39590327 PMC11593303

[121] Antony P Vijayan R. Bioactive peptides as potential nutraceuticals for diabetes therapy: a comprehensive review. Int J Mol Sci. (2021) 22:9059. doi: 10.3390/ijms22169059, PMID: 34445765 PMC8396489

[122] Rivero-Pino F Espejo-Carpio FJ Guadix EM. Antidiabetic food-derived peptides for functional feeding: production, functionality and in vivo evidence. Food Secur. (2020) 9:983. doi: 10.3390/foods9080983, PMID: 32718070 PMC7466190

[123] Zhang Q Wu C Wang T Sun Y Li T Fan G. Improvement of biological activity of Morchella esculenta protein hydrolysate by microwave-assisted Selenization. J Food Sci. (2019) 84:73–9. doi: 10.1111/1750-3841.14411, PMID: 30575032

[124] Lammi C Zanoni C Arnoldi A Vistoli G. Peptides derived from soy and Lupin protein as dipeptidyl-peptidase IV inhibitors: in vitro biochemical screening and in silico molecular modeling study. J Agric Food Chem. (2016) 64:9601–6. doi: 10.1021/acs.jafc.6b04041, PMID: 27983830

[125] Mojica L Luna-Vital DA González de Mejía E. Characterization of peptides from common bean protein isolates and their potential to inhibit markers of type-2 diabetes, hypertension and oxidative stress. J Sci Food Agric. (2017) 97:2401–10. doi: 10.1002/jsfa.8053, PMID: 27664971

[126] Wang Y Liu Y Wang H Li C Qi P Bao J. Agaricus bisporus lectins mediates islet β-cell proliferation through regulation of cell cycle proteins. Exp Biol Med. (2012) 237:287–96. doi: 10.1258/ebm.2011.011251, PMID: 22393165

[127] Sousa AS Araújo-Rodrigues H Pintado ME. The health-promoting potential of edible mushroom proteins. Curr Pharm Des. (2022) 29:804–23. doi: 10.2174/1381612829666221223103756 36567303

[128] Liu Z Gong J Huang W Lu F Dong H. The effect of *Momordica charantia* in the treatment of diabetes mellitus: a review. Evid Based Complement Alternat Med. (2021) 2021:3796265. doi: 10.1155/2021/3796265, PMID: 33510802 PMC7826218

[129] Richter E Geetha T Burnett D Broderick TL Babu JR. The effects of *Momordica charantia* on type 2 diabetes mellitus and Alzheimer’s disease. Int J Mol Sci. (2023) 24:4643. doi: 10.3390/ijms24054643, PMID: 36902074 PMC10002567

[130] Xu B Li Z Zeng T Zhan J Wang S Ho CT. Bioactives of *Momordica charantia* as potential anti-diabetic/hypoglycemic agents. Molecules. (2022) 27:1–17. doi: 10.3390/molecules27072175 PMC9000558 35408574

[131] Peter EL Mtewa AG Nagendrappa PB Kaligirwa A Sesaazi CD. Systematic review and meta-analysis protocol for efficacy and safety of *Momordica charantia* L. on animal models of type 2 diabetes mellitus. Syst Rev. (2020) 9:1–9. doi: 10.1186/s13643-019-1265-4 31915054 PMC6950794

[132] Oyelere SF Ajayi OH Ayoade TE Santana Pereira GB Dayo Owoyemi BC Ilesanmi AO. A detailed review on the phytochemical profiles and anti-diabetic mechanisms of *Momordica charantia*. Heliyon. (2022) 8:e09253. doi: 10.1016/j.heliyon.2022.e09253, PMID: 35434401 PMC9010624

[133] Salleh NH Zulkipli IN Mohd Yasin H Ja’Afar F Ahmad N Wan Ahmad WAN. Systematic review of medicinal plants used for treatment of diabetes in human clinical trials: an ASEAN perspective. Evid Based Complement Alternat Med. (2021) 2021:5570939. doi: 10.1155/2021/5570939, PMID: 34691218 PMC8528580

[134] Jane-Frances OA Chichaya TF. The effectiveness and safety of bitter melon in the management of type 2 diabetes mellitus: a review of randomized controlled trials. Integr Complement Ther. (2023) 29:277–85. doi: 10.1089/ict.2023.29107.oaj

[135] Visuvanathan T Than LTL Stanslas J Chew SY Vellasamy S. Revisiting *Trigonella foenum-graecum* L.: pharmacology and therapeutic potentialities. Plan Theory. (2022) 11:1–14. doi: 10.3390/plants11111450 PMC9182856 35684222

[136] Giuntini EB Sardá FAH de Menezes EW. The effects of soluble dietary fibers on glycemic response: an overview and futures perspectives. Food Secur. (2022) 11:1–26. doi: 10.3390/foods11233934 PMC9736284 36496742

[137] Avalos-Soriano A De La Cruz-Cordero R Rosado JL Garcia-Gasca T. 4-hydroxyisoleucine from fenugreek (*Trigonella foenum-graecum*): effects on insulin resistance associated with obesity. Molecules. (2016) 21:1–12. doi: 10.3390/molecules21111596 PMC6273931 27879673

[138] Hamden K Mnafgui K Amri Z Aloulou A Elfeki A. Inhibition of key digestive enzymes related to diabetes and hyperlipidemia and protection of liver-kidney functions by trigonelline in diabetic rats. Sci Pharm. (2013) 81:233–46. doi: 10.3797/scipharm.1211-14, PMID: 23641341 PMC3617660

[139] Niu Y Niu H Chi L Li P Du J Wang X. *Trigonella foenum-graecum* L. protects against renal function decline in a mouse model of type 2 diabetic nephropathy by modulating the PI3K-Akt-ERK signaling pathway. Front Pharmacol. (2025) 16:1–20. doi: 10.3389/fphar.2025.1566723 PMC11959092 40170727

[140] Sharma S Choudhary M Budhwar V. Role of bioactive phytoconstituents as modulators of hepatic carbohydrates metabolising enzymes: a target specific approach to treat diabetes mellitus. Curr Diabetes Rev. (2022) 18:57–72. doi: 10.2174/1573399818666220210140745 35142270

[141] Gauttam VK Munjal K Chopra H Ahmad A Rana MK Kamal MA. A mechanistic review on therapeutic potential of medicinal plants and their pharmacologically active molecules for targeting metabolic syndrome. Curr Pharm Des. (2024) 30:10–30. doi: 10.2174/0113816128274446231220113957, PMID: 38155468

[142] Semwal DK Kumar A Aswal S Chauhan A Semwal RB. Protective and therapeutic effects of natural products against diabetes mellitus via regenerating pancreatic β-cells and restoring their dysfunction. Phytother Res. (2021) 35:1218–29. doi: 10.1002/ptr.6885, PMID: 32987447

[143] Akbari S Abdurahman NH Yunus RM Alara OR Abayomi OO. Extraction, characterization and antioxidant activity of fenugreek (*Trigonella-Foenum graecum*) seed oil. Mater Sci Energy Technol. (2019) 2:349–55. doi: 10.1016/J.MSET.2018.12.001

[144] Chehregosha F Maghsoumi-Norouzabad L Mobasseri M Fakhr L Tarighat-Esfanjani A. The effect of fenugreek seed dry extract supplement on glycemic indices, lipid profile, and prooxidant-antioxidant balance in patients with type 2 diabetes: a double-blind randomized clinical trial. J Cardiovasc Thorac Res. (2024) 16:184–93. doi: 10.34172/jcvtr.33231, PMID: 39430281 PMC11489642

[145] Islam J Islam Z Haque N Khatun M Islam F Hossain S. Fenugreek seed powder protects mice against arsenic-induced neurobehavioral changes. Curr Res Toxicol. (2023) 5:100114. doi: 10.1016/J.CRTOX.2023.100114, PMID: 37554151 PMC10404539

[146] Tomlinson B Patil NG Fok M Lam CWK. Managing dyslipidemia in patients with type 2 diabetes. Expert Opin Pharmacother. (2021) 22:2221–34. doi: 10.1080/14656566.2021.1912734, PMID: 33823719

[147] Gavahian M Bannikoppa AM Majzoobi M Hsieh CW Lin J Farahnaky A. Fenugreek bioactive compounds: a review of applications and extraction based on emerging technologies. Crit Rev Food Sci Nutr. (2024) 64:10187–203. doi: 10.1080/10408398.2023.2221971, PMID: 37303155

[148] Aylanc V Eskin B Zengin G Dursun M Cakmak YS. In vitro studies on different extracts of fenugreek (*Trigonella spruneriana* BOISS.): phytochemical profile, antioxidant activity, and enzyme inhibition potential. J Food Biochem. (2020) 44:1–10. doi: 10.1111/jfbc.13463 32931607

[149] Mansoori A Hosseini S Zilaee M Hormoznejad R Fathi M. Effect of fenugreek extract supplement on testosterone levels in male: a meta-analysis of clinical trials. Phytother Res. (2020) 34:1550–5. doi: 10.1002/PTR.6627, PMID: 32048383

[150] Janda K Gutowska I Geszke-Moritz M Jakubczyk K. The common cichory (*Cichorium intybus* L.) as a source of extracts with health-promoting properties—a review. Molecules. (2021) 26:1–14. doi: 10.3390/molecules26061814 PMC8005178 33807029

[151] Birsa ML Sarbu LG. Health benefits of key constituents in *Cichorium intybus* L. Nutrients. (2023) 15:1322. doi: 10.3390/nu15061322, PMID: 36986053 PMC10058675

[152] Khan AMA Chandra K. “Medicinal and nutritional importance of *Cichorium intybus* in human health,” In: Ansari MA Shoaib S Islam N, editors. Medicinal plants and their bioactive compounds in human health Vol 1, Singapore: Springer Nature Singapore Pte Ltd. (2024) 251–71.

[153] Nasimi Doost Azgomi R Karimi A Tutunchi H Moini Jazani A. A comprehensive mechanistic and therapeutic insight into the effect of chicory (*Cichorium intybus*) supplementation in diabetes mellitus: a systematic review of literature. Int J Clin Pract. (2021) 75:e14945. doi: 10.1111/ijcp.14945, PMID: 34606165

[154] Kt RD Sivalingam N. *Cichorium intybus* attenuates streptozotocin-induced pancreatic β-cell damage by inhibiting NF-κB activation and oxidative stress. J Appl Biomed. (2020) 18:70–9. doi: 10.32725/jab.2020.010, PMID: 34907728

[155] Ebrahiminia M Esmaeili F Shabani L. In vitro differentiation induction of embryonal carcinoma stem cells into insulin-producing cells by *Cichorium intybus* L. leaf extract. J Ethnopharmacol. (2020) 246:112214. doi: 10.1016/J.JEP.2019.112214, PMID: 31491437

[156] Krepkova LV Babenko AN Lemyaseva SV Saybel OL Sherwin CM Enioutina EY. Modulation of hepatic functions by chicory (*Cichorium intybus* L.) extract: preclinical study in rats †. Pharmaceuticals. (2023) 16:1471. doi: 10.3390/ph16101471, PMID: 37895942 PMC10609820

[157] Singh M Rani H Chopra HK. Extraction, optimization, purification and characterization of inulin from chicory roots using conventional and greener extraction techniques. Int J Biol Macromol. (2025) 306:141385. doi: 10.1016/J.IJBIOMAC.2025.141385, PMID: 39988165

[158] El-Kholy WM Aamer RA Ali ANA. Utilization of inulin extracted from chicory (*Cichorium intybus* L.) roots to improve the properties of low-fat synbiotic yoghurt. Ann Agric Sci. (2020) 65:59–67. doi: 10.1016/J.AOAS.2020.02.002

[159] Guo W Yun J Wang B Xu S Ye C Wang X. Comparative study on physicochemical properties and hypoglycemic activities of intracellular and extracellular polysaccharides from submerged fermentation of Morchella esculenta. Int J Biol Macromol. (2024) 278:134759. doi: 10.1016/j.ijbiomac.2024.134759, PMID: 39151842

[160] Rehman AU Siddiqui NZ Farooqui NA Alam G Gul A Ahmad B. Morchella esculenta mushroom polysaccharide attenuates diabetes and modulates intestinal permeability and gut microbiota in a type 2 diabetic mice model. Front Nutr. (2022) 9:984695. doi: 10.3389/fnut.2022.984695, PMID: 36276816 PMC9582931

[161] Wu H Chen J Liu Y Cheng H Nan J Park HJ. Digestion profile, antioxidant, and antidiabetic capacity of *Morchella esculenta* exopolysaccharide: in vitro, in vivo and microbiota analysis. J Sci Food Agric. (2023) 103:4401–12. doi: 10.1002/jsfa.12513, PMID: 36807912

[162] Shurong W Dongjie L Guangle L Naixin D Chang H Junlong M. Functional properties, rheological characteristics, simulated digestion, and fermentation by human fecal microbiota of polysaccharide from *Morchella importuna*. Food Secur. (2024) 13:1–20. doi: 10.3390/foods13132148 PMC11241200 38998652

[163] Thaiss CA Levy M Grosheva I Zheng D Soffer E Blacher E. Hyperglycemia drives intestinal barrier dysfunction and risk for enteric infection. Science. (2018) 359:1376–83. doi: 10.1126/science.aar3318, PMID: 29519916

[164] Begum N Nasir A Parveen Z Muhammad T Ahmed A Farman S. Evaluation of the hypoglycemic activity of Morchella conica by targeting protein tyrosine phosphatase 1B. Front Pharmacol. (2021) 12:661803. doi: 10.3389/fphar.2021.661803, PMID: 34093192 PMC8173442

[165] Hussain H Green IR Abbas G Adekenov SM Hussain W Ali I. Protein tyrosine phosphatase 1B (PTP1B) inhibitors as potential anti-diabetes agents: patent review (2015–2018). Expert Opin Ther Pat. (2019) 29:689–702. doi: 10.1080/13543776.2019.1655542, PMID: 31402706

[166] Cui W Song X Li X Jia L Zhang C. Structural characterization of Hericium erinaceus polysaccharides and the mechanism of anti-T2DM by modulating the gut microbiota and metabolites. Int J Biol Macromol. (2023) 242:125165. doi: 10.1016/J.IJBIOMAC.2023.125165, PMID: 37270132

[167] Lee SK Ryu SH Turk A Yeon SW Jo YH Han YK. Characterization of α-glucosidase inhibitory constituents of the fruiting body of lion’s mane mushroom (Hericium erinaceus). J Ethnopharmacol. (2020) 262:113197. doi: 10.1016/j.jep.2020.113197, PMID: 32738392

[168] Cui J Chisti Y. Polysaccharopeptides of Coriolus versicolor: physiological activity, uses, and production. Biotechnol Adv. (2003) 21:109–22. doi: 10.1016/S0734-9750(03)00002-8, PMID: 14499133

[169] Su CH Lai MN Ng LT. Inhibitory effects of medicinal mushrooms on α-amylase and α-glucosidase – enzymes related to hyperglycemia. Food Funct. (2013) 4:644–9. doi: 10.1039/c3fo30376d, PMID: 23396484

[170] Teng JF Lee CH Hsu TH Lo HC. Potential activities and mechanisms of extracellular polysaccharopeptides from fermented Trametes versicolor on regulating glucose homeostasis in insulin-resistant HepG2 cells. PLoS One. (2018) 13:e0201131–18. doi: 10.1371/journal.pone.0201131, PMID: 30024975 PMC6053205

[171] Furman BL. Streptozotocin-induced diabetic models in mice and rats. Curr Protoc Pharmacol. (2021) 1:1–21. doi: 10.1002/cpz1.78 26331889

[172] Lo H-C Hsu T-H Lee C-H. Extracellular polysaccharopeptides from fermented Turkey tail medicinal mushroom, *Trametes versicolor* (Agaricomycetes), mitigate oxidative stress, hyperglycemia, and hyperlipidemia in rats with type 2 diabetes mellitus. Int J Med Mushrooms. (2020) 22:417–29. doi: 10.1615/IntJMedMushrooms.2020034560, PMID: 32749097

[173] Xian HM Che H Qin Y Yang F Meng SY Li XG. *Coriolus versicolor* aqueous extract ameliorates insulin resistance with PI3K/Akt and p38 MAPK signaling pathways involved in diabetic skeletal muscle. Phytother Res. (2018) 32:551–60. doi: 10.1002/ptr.6007, PMID: 29243310

[174] Wang Y Li H Li Y Zhao Y Xiong F Liu Y. *Coriolus versicolor* alleviates diabetic cardiomyopathy by inhibiting cardiac fibrosis and NLRP3 inflammasome activation. Phytother Res. (2019) 33:2737–48. doi: 10.1002/ptr.6448 31338905

[175] Meng F Lin Y Hu L Feng W Su P Wu L. The therapeutic effect of Coriolus versicolor fruiting body on STZ-induced ICR diabetic mice. J Healthc Eng. (2022) 2022:1–9. doi: 10.1155/2022/7282453, PMID: 35463673 PMC9033387

[176] Chen C-H Kang L Lo H-C Hsu T-H Lin F-Y Shan L y. Polysaccharides of *Trametes versicolor* improve bone properties in diabetic rats. J Agric Food Chem. (2015) 63:9232-8. doi: 10.1021/acs.jafc.5b02668 26308886

[177] Shimokawa K Mashima I Asai A Yamada K Kita M Uemura D. (−)-Ternatin, a highly N-methylated cyclic heptapeptide that inhibits fat accumulation: structure and synthesis. Tetrahedron Lett. (2006) 26:4445–8.

[178] Shimokawa K Iwase Y Yamada K Uemura D. Synthesis and inhibitory effect on fat accumulation of (−)-ternatin derivatives modified in the β-OH-d-Leu7 moiety. Org Biomol Chem. (2008) 6:58–60. doi: 10.1039/b714710d 18075648

[179] Kobayashi M Kawashima H Takemori K Ito H Murai A Masuda S. Ternatin, a cyclic peptide isolated from mushroom, and its derivative suppress hyperglycemia and hepatic fatty acid synthesis in spontaneously diabetic KK-ay mice. Biochem Biophys Res Commun. (2012) 427:299–304. doi: 10.1016/j.bbrc.2012.09.045, PMID: 23000156

[180] Tomšik A Pavlić B Vladić J Ramić M Brindza J Vidović S. Optimization of ultrasound-assisted extraction of bioactive compounds from wild garlic (*Allium ursinum* L.). Ultrason Sonochem. (2016) 29:502–11. doi: 10.1016/j.ultsonch.2015.11.005, PMID: 26563916

[181] Cvetanović Kljakić A Stupar A Terzić M Božunović J Gašić U Zengin G. Chemical profiling and biological activities of Opopanax hispidus extracts: a comparative insight on conventional and green extraction technologies. Sustain Chem Pharm. (2023) 33:101122. doi: 10.1016/J.SCP.2023.101122, PMID: 40399227

[182] Belwal T Chemat F Venskutonis PR Cravotto G Jaiswal DK Bhatt ID. Recent advances in scaling-up of non-conventional extraction techniques: learning from successes and failures. TrAC Trends Anal Chem. (2020) 127:115895. doi: 10.1016/J.TRAC.2020.115895

[183] Alañón ME Ivanović M Pimentel-Mora S Borrás-Linares I Arráez-Román D Segura-Carretero A. A novel sustainable approach for the extraction of value-added compounds from *Hibiscus sabdariffa* L. calyces by natural deep eutectic solvents. Food Res Int. (2020) 137:109646. doi: 10.1016/j.foodres.2020.109646, PMID: 33233225

[184] Pojić M Teslić N Banjac V Mrkonjić Ž Stupar A Mandić A. Natural deep eutectic solvents for efficient recovery of bioactive compounds from by-product of industrial hemp processing: pretreatment, modeling and optimization. Ind Crop Prod. (2024) 222:119617. doi: 10.1016/j.indcrop.2024.119617

[185] Teslić N Santos F Oliveira F Stupar A Pojić M Mandić A. Simultaneous hydrolysis of ellagitannins and extraction of ellagic acid from defatted raspberry seeds using natural deep eutectic solvents (NADES). Antioxidants. (2022) 11:254. doi: 10.3390/antiox11020254 35204137 PMC8868079

[186] Barbieri R Coppo E Marchese A Daglia M Sobarzo-Sánchez E Nabavi SF. Phytochemicals for human disease: an update on plant-derived compounds antibacterial activity. Microbiol Res. (2017) 196:44–68. doi: 10.1016/J.MICRES.2016.12.003, PMID: 28164790

[187] Khan MF Rawat AK Khatoon S Hussain MK Mishra A Negi DS. In vitro and in vivo antidiabetic effect of extracts of *Melia azedarach*, Zanthoxylum alatum, and Tanacetum nubigenum. Integr Med Res. (2018) 7:176–83. doi: 10.1016/J.IMR.2018.03.004, PMID: 29984178 PMC6026355

[188] Sut S Dall’Acqua S Zengin G Senkardes I Bulut G Cvetanović A. Influence of different extraction techniques on the chemical profile and biological properties of *Anthemis cotula* L.: multifunctional aspects for potential pharmaceutical applications. J Pharm Biomed Anal. (2019) 173:75–85. doi: 10.1016/J.JPBA.2019.05.028 31121457

[189] Nayak B Dahmoune F Moussi K Remini H Dairi S Aoun O. Comparison of microwave, ultrasound and accelerated-assisted solvent extraction for recovery of polyphenols from *Citrus sinensis* peels. Food Chem. (2015) 187:507–16. doi: 10.1016/j.foodchem.2015.04.081, PMID: 25977057

[190] Cheung LM Cheung PCK. Mushroom extracts with antioxidant activity against lipid peroxidation. Food Chem. (2005) 89:403–9. doi: 10.1016/J.FOODCHEM.2004.02.049

[191] Leong YK Yang FC Chang JS. Extraction of polysaccharides from edible mushrooms: emerging technologies and recent advances. Carbohydr Polym. (2021) 251:117006. doi: 10.1016/J.CARBPOL.2020.117006, PMID: 33142573

[192] Chen Y Gu X Huang S q Li J Wang X Tang J. Optimization of ultrasonic/microwave assisted extraction (UMAE) of polysaccharides from *Inonotus obliquus* and evaluation of its anti-tumor activities. Int J Biol Macromol. (2010) 46:429–35. doi: 10.1016/J.IJBIOMAC.2010.02.003, PMID: 20149817

[193] Smiderle FR Morales D Gil-Ramírez A de Jesus LI Gilbert-López B Iacomini M. Evaluation of microwave-assisted and pressurized liquid extractions to obtain β-d-glucans from mushrooms. Carbohydr Polym. (2017) 156:165–74. doi: 10.1016/J.CARBPOL.2016.09.029, PMID: 27842810

[194] Almeida CF Manrique YA Lopes JCB Martins FG Dias MM. Recovery of ergosterol from Agaricus bisporus mushrooms via supercritical fluid extraction: a response surface methodology optimisation. Heliyon. (2024) 10:e21943. doi: 10.1016/J.HELIYON.2023.E21943, PMID: 39676796 PMC11639697

[195] Milovanovic I Zengin G Maksimovic S Tadic V. Supercritical and ultrasound-assisted extracts from Pleurotus pulmonarius mushroom: chemical profiles, antioxidative, and enzyme-inhibitory properties. J Sci Food Agric. (2021) 101:2284–93. doi: 10.1002/jsfa.10849, PMID: 33006768

[196] Milovanovic I Zengin G Maksimovic S Tadic V. Supercritical carbon-oxide extracts from cultivated and wild-grown Ganoderma lucidum mushroom: differences in ergosterol and ganoderic acids content, antioxidative and enzyme inhibitory properties. Nat Prod Res. (2024) 38:2522–8. doi: 10.1080/14786419.2023.2175355, PMID: 36744699

[197] Farooq S Shah MA Siddiqui MW Dar BN Mir SA Ali A. Recent trends in extraction techniques of anthocyanins from plant materials. J Food Meas Charact. (2020) 14:3508–19. doi: 10.1007/s11694-020-00598-8

[198] Cvetanović Kljakić A Božunović J Gašić U Seebaluck-Sandoram R Uba AI Mahomoodally MF. Chemical characterization of *Glaucosciadum cordifolium* extracts obtained by different extraction techniques and their biopharmaceutical effects. Process Biochem. (2023) 134:141–50. doi: 10.1016/J.PROCBIO.2023.10.007

[199] Duran T Peron G Zancato M Zengin G Cetiz MV Bouyahya A. Harnessing the chemical composition and anti-oxidant, anti-enzymatic, and anti-cancer activities of two Corydalis species (*C. Erdelii* and *C. solida*) by using in vitro and in silico analysis. Food Biosci. (2024) 61:104762. doi: 10.1016/J.FBIO.2024.104762

[200] World Health Organization (WHO). International Regulatory Cooperation for Herbal Medicines (IRCH). Available online at: https://www.who.int/initiatives/international-regulatory-cooperation-for-herbal-medicines (Accessed June 26, 2024).

[201] Bouknana S Lafdil FZ Kandsi F Driouech M Conte R Bouknana D. Antidiabetic and aldose reductase inhibitory activity and LC-MS/MS compositional polyphenol determination of aqueous extract of *Ammodaucus leucotrichus* fruits. Biocatal Agric Biotechnol. (2024) 58:103100. doi: 10.1016/J.BCAB.2024.103100

[202] Ahmad S Ahmad MFA Alouffi S Khan S Khan M Khan MWA. Aldose reductase inhibitory and antiglycation properties of phytoconstituents of *Cichorium intybus*: potential therapeutic role in diabetic retinopathy. Int J Biol Macromol. (2024) 277:133816. doi: 10.1016/J.IJBIOMAC.2024.133816, PMID: 39002911

[203] Kundur S Shyam P. Quantification of total phenols in *Coleus forskohlii* and inhibition of aldose reductase by rosmarinic acid. J Mol Struct. (2024) 1312:138435. doi: 10.1016/J.MOLSTRUC.2024.138435

[204] Nebrigić V Terzić M Đurović S Micić D Zengin G Kljakić AC. Influence of drying process on chemical composition, antioxidant and enzyme-inhibitory activity of *Helichrysum italicum* essential oils. J Herb Med. (2023) 40:100680. doi: 10.1016/j.hermed.2023.100680

[205] Diamantopoulou P Philippoussis A. “Cultivated mushrooms: preservation and processing,” In: Sinha N Hui YH Evranuz EÖ Siddiq M Ahmed J, editors. Handbook of vegetable preservation and processing. Boca Raton, FL, United States: CRC Press (2015). 495–525.

[206] Dilshad R Khan K u R Dilshad R Ahmad S Rao H Khurshid U. Comprehensive chemical profiling with UHPLC-MS, in-vitro, in-silico, and in-vivo antidiabetic potential of *Typha domingensis* Pers; a novel source of bioactive compounds. S Afr J Bot. (2024) 171:185–98. doi: 10.1016/J.SAJB.2024.06.007

[207] Zolotova D Teterovska R Bandere D Lauberte L Niedra S. Antidiabetic properties of the root extracts of dandelion (*Taraxacum officinale*) and burdock (*Arctium lappa*). Plan Theory. (2024) 13:1021. doi: 10.3390/PLANTS13071021, PMID: 38611548 PMC11013470

[208] Mechchate H Es-Safi I Louba A Alqahtani AS Nasr FA Noman OM. In vitro alpha-amylase and alpha-glucosidase inhibitory activity and in vivo antidiabetic activity of Withania frutescens L. foliar extract. Molecules. (2021) 26:293. doi: 10.3390/MOLECULES26020293 33430115 PMC7826620

[209] Bljajić K Petlevski R Vujić L Čačić A Šoštarić N Jablan J. Chemical composition, antioxidant and α-glucosidase-inhibiting activities of the aqueous and hydroethanolic extracts of *Vaccinium myrtillus* leaves. Molecules. (2017) 22:703. doi: 10.3390/MOLECULES22050703 28452948 PMC6154652

[210] Rodrigues MJ Custódio L Lopes A Oliveira M Neng NR Nogueira JMF. Unlocking the in vitro anti-inflammatory and antidiabetic potential of *Polygonum maritimum*. Pharm Biol. (2017) 55:1348–57. doi: 10.1080/13880209.2017.1301493, PMID: 28301958 PMC6130642

[211] Shettar AK Sateesh MK Kaliwal BB Vedamurthy AB. In vitro antidiabetic activities and GC-MS phytochemical analysis of *Ximenia americana* extracts. S Afr J Bot. (2017) 111:202–11. doi: 10.1016/J.SAJB.2017.03.014

[212] Justino AB Miranda NC Franco RR Martins MM Silva NM da Espindola FS. *Annona muricata* Linn. Leaf as a source of antioxidant compounds with in vitro antidiabetic and inhibitory potential against α-amylase, α-glucosidase, lipase, non-enzymatic glycation and lipid peroxidation. Biomed Pharmacother (2018) 100:83–92. doi: 10.1016/J.BIOPHA.2018.01.172 29425747

[213] Soni LK Dobhal MP Arya D Bhagour K Parasher P Gupta RS. In vitro and in vivo antidiabetic activity of isolated fraction of *Prosopis cineraria* against streptozotocin-induced experimental diabetes: a mechanistic study. Biomed Pharmacother. (2018) 108:1015–21. doi: 10.1016/J.BIOPHA.2018.09.099, PMID: 30372801

[214] Hu XF Zhang Q Zhang PP Sun LJ Liang JC Morris-Natschke SL. Evaluation of in vitro/in vivo anti-diabetic effects and identification of compounds from *Physalis alkekengi*. Fitoterapia. (2018) 127:129–37. doi: 10.1016/J.FITOTE.2018.02.015, PMID: 29447981

[215] Siddique MH Ashraf A Hayat S Aslam B Fakhar-e-Alam M Muzammil S. Antidiabetic and antioxidant potentials of *Abelmoschus esculentus*: in vitro combined with molecular docking approach. J Saudi Chem Soc. (2022) 26:101418. doi: 10.1016/J.JSCS.2021.101418

[216] Wei Q Zhan Y Chen B Xie B Fang T Ravishankar S. Assessment of antioxidant and antidiabetic properties of Agaricus blazei Murill extracts. Food Sci Nutr. (2020) 8:332–9. doi: 10.1002/fsn3.1310, PMID: 31993159 PMC6977522

[217] Ratnaningtyas NI Hernayanti H Ekowati N Husen F. Ethanol extract of the mushroom Coprinus comatus exhibits antidiabetic and antioxidant activities in streptozotocin-induced diabetic rats. Pharm Biol. (2022) 60:1126–36. doi: 10.1080/13880209.2022.2074054, PMID: 35675226 PMC9186368

[218] Gao Z Kong D Cai W Zhang J Jia L. Characterization and anti-diabetic nephropathic ability of mycelium polysaccharides from Coprinus comatus. Carbohydr Polym. (2021) 251:117081. doi: 10.1016/j.carbpol.2020.117081, PMID: 33142624

[219] Wang H Yang Y Wang S Li C Chen C Wan X. Polysaccharides of *Floccularia luteovirens* alleviate oxidative damage and inflammatory parameters of diabetic nephropathy in db/db mice. Front Biosci (Landmark Ed). (2023) 28:1–10. doi: 10.31083/j.fbl2804082, PMID: 37114539

[220] Keshavarz-Rezaei M Hatamian-Zarmi A Alvandi H Ebrahimi-Hosseinzadeh B Mokhtari-Hosseini ZB. The HbA1c and blood glucose response to selenium-rich polysaccharide from Fomes fomentarius loaded solid lipid nanoparticles as a potential antidiabetic agent in rats. Biomater Adv. (2022) 140:213084. doi: 10.1016/J.BIOADV.2022.213084, PMID: 36027667

[221] Hossain MS Barua A Tanim MAH Hasan MS Islam MJ Hossain MR. Ganoderma applanatum mushroom provides new insights into the management of diabetes mellitus, hyperlipidemia, and hepatic degeneration: a comprehensive analysis. Food Sci Nutr. (2021) 9:4364–74. doi: 10.1002/fsn3.2407, PMID: 34401085 PMC8358375

[222] Teng BS Wang CD Yang HJ Wu JS Zhang D Zheng M. A protein tyrosine phosphatase 1B activity inhibitor from the fruiting bodies of Ganoderma lucidum (Fr.) karst and its hypoglycemic potency on streptozotocin-induced type 2 diabetic mice. J Agric Food Chem. (2011) 59:6492–500. doi: 10.1021/jf200527y, PMID: 21585203

[223] Xiao C Wu Q Zhang J Xie Y Cai W Tan J. Antidiabetic activity of Ganoderma lucidum polysaccharides F31 down-regulated hepatic glucose regulatory enzymes in diabetic mice. J Ethnopharmacol. (2017) 196:47–57. doi: 10.1016/j.jep.2016.11.044, PMID: 27902927

[224] Feng X Wang P Lu Y Zhang Z Yao C Tian G. A novel polysaccharide from Heimioporus retisporus displays hypoglycemic activity in a diabetic mouse model. Front Nutr. (2022) 9:964948. doi: 10.3389/fnut.2022.964948, PMID: 35898716 PMC9311259

[225] Liu Q Wu J Wang P Lu Y Ban X. Neutral polysaccharides from *Hohenbuehelia serotina* with hypoglycemic effects in a type 2 diabetic mouse model. Front Pharmacol. (2022) 13:1–10. doi: 10.3389/fphar.2022.883653 PMC9117631 35600885

[226] Ye X Wu K Xu L Cen Y Ni J Chen J. Methanol extract of Inonotus obliquus improves type 2 diabetes mellitus through modifying intestinal flora. Front Endocrinol (Lausanne). (2023) 13:1103972. doi: 10.3389/fendo.2022.1103972, PMID: 36686454 PMC9852891

[227] Wunjuntuk K Ahmad M Techakriengkrai T Chunhom R Jaraspermsuk E Chaisri A. Proximate composition, dietary fibre, beta-glucan content, and inhibition of key enzymes linked to diabetes and obesity in cultivated and wild mushrooms. J Food Compos Anal. (2022) 105:104226. doi: 10.1016/J.JFCA.2021.104226

[228] Gong P Wang X Liu M Wang M Wang S Guo Y. Hypoglycemic effect of a novel polysaccharide from Lentinus edodes on STZ-induced diabetic mice via metabolomics study and Nrf2/HO-1 pathway. Food Funct. (2022) 13:3036–49. doi: 10.1039/d1fo03487a, PMID: 35199807

[229] Hussein A Ghonimy A Jiang H Qin G El-Ashram S Hussein S. LC/MS analysis of mushrooms provided new insights into dietary management of diabetes mellitus in rats. Food Sci Nutr. (2023) 11:2321–35. doi: 10.1002/fsn3.3236, PMID: 37181306 PMC10171545

[230] Uzomba CG Ezemagu UK Ofoegbu M-S Lydia N Goodness E Emelike C. Edible mushroom (Pleurotus cornucopiae) extract vs. glibenclamide on alloxan induced diabetes: sub-acute in vivo study of Nrf2 expression and renal toxicity. Anat Cell Biol. (2024) 57:446–58. doi: 10.5115/acb.24.054, PMID: 38972671 PMC11424557

[231] Azeem U Shri R Dhingra GS. In vitro and in vivo Antihyperglycemic activities of medicinal mushrooms (Agaricomycetes) from India. Int J Med Mushrooms. (2021) 23:29–41. doi: 10.1615/IntJMedMushrooms.2021037630, PMID: 33639079

[232] Wang X y Jiang S Liu Y. Anti-diabetic effects of fungal ergosta-4, 6, 8(14), 22-tetraen-3-one from *Pholiota adiposa*. Steroids. (2023) 192:109185. doi: 10.1016/J.STEROIDS.2023.109185, PMID: 36720423

[233] Çayan F Tel-Çayan G Deveci E Duru ME. HPLC–DAD characterization of phenolic profile and in vitro antioxidant, anticholinesterase, and antidiabetic activities of five mushroom species from Turkey. 3 Biotech. (2021) 11:273. doi: 10.1007/s13205-021-02819-3, PMID: 34055565 PMC8128964

